# Biofilms and core pathogens shape the tumor microenvironment and immune phenotype in colorectal cancer

**DOI:** 10.1080/19490976.2024.2350156

**Published:** 2024-05-10

**Authors:** Lasse Kvich, Blaine Gabriel Fritz, Henrike Zschach, Thilde Terkelsen, Hans Raskov, Kathrine Høst-Rasmussen, Morten Ragn Jakobsen, Alexandra Gabriella Gheorghe, Ismail Gögenur, Thomas Bjarnsholt

**Affiliations:** a Center for Surgical Science, Department of Surgery, Zealand University Hospital, Region Zealand, Denmark; b Costerton Biofilm Center, Department of Immunology and Microbiology, University of Copenhagen, Copenhagen, Denmark; c Center for Health Data Science, University of Copenhagen, Copenhagen, Denmark; d Department of Forensic Medicine, Faculty of Health and Medical Sciences, University of Copenhagen, Copenhagen, Denmark; e Department of Clinical Medicine, University of Copenhagen, Copenhagen, Denmark; f Department of Clinical Microbiology, Rigshospitalet, Copenhagen, Denmark

**Keywords:** Colorectal cancer (CRC), biofilms, *Fusobacterium nucleatum*, *Bacteroides fragilis*, *in situ* hybridization, fluorescence, sequence analysis, RNA

## Abstract

Extensive research has explored the role of gut microbiota in colorectal cancer (CRC). Nonetheless, metatranscriptomic studies investigating the *in situ* functional implications of host-microbe interactions in CRC are scarce. Therefore, we characterized the influence of CRC core pathogens and biofilms on the tumor microenvironment (TME) in 40 CRC, paired normal, and healthy tissue biopsies using fluorescence *in situ* hybridization (FISH) and dual-RNA sequencing. FISH revealed that *Fusobacterium spp*. was associated with increased bacterial biomass and inflammatory response in CRC samples. Dual-RNA sequencing demonstrated increased expression of pro-inflammatory cytokines, defensins, matrix-metalloproteases, and immunomodulatory factors in CRC samples with high bacterial activity. In addition, bacterial activity correlated with the infiltration of several immune cell subtypes, including M2 macrophages and regulatory T-cells in CRC samples. Specifically, *Bacteroides fragilis* and *Fusobacterium nucleatum* correlated with the infiltration of neutrophils and CD4^+^ T-cells, respectively. The collective bacterial activity/biomass appeared to exert a more significant influence on the TME than core pathogens, underscoring the intricate interplay between gut microbiota and CRC. These results emphasize how biofilms and core pathogens shape the immune phenotype and TME in CRC while highlighting the need to extend the bacterial scope beyond CRC pathogens to advance our understanding and identify treatment targets.

## Introduction

Several studies demonstrate distinct gut microbiota compositions in patients with colorectal cancer (CRC), highlighting the enrichment of specific species.^1–3^ This imbalance of the gut microbiota is termed microbial dysbiosis and is thought to contribute to CRC pathogenesis.^4,5^ Microbial dysbiosis allows opportunistic bacteria, such as *Bacteroides fragilis* and *Fusobacterium nucleatum*, to accumulate and increase inflammation in the tissue – a known risk factor for CRC.^6,7^ Both species are frequently enriched in CRC tissue, and multiple *in vitro* and *in vivo* experiments have explored the mechanisms by which these core pathogens accelerate carcinogenesis.^8–13^ However, mechanistic insights from animal experiments or *in vitro* studies do not adequately characterize the *in situ* interplay between bacteria and the host.^14^ While *in vitro* models or animal studies focusing on specific bacterial species may aid in understanding CRC-inducing mechanisms, CRC progression and CRC-related inflammation likely involve diverse bacterial, fungal, and viral constellations.^3,15^ In other words, these models fall short of accurately representing the complex *in vivo* tumor microenvironment (TME), where both pro- and anti-tumorigenic mechanisms operate simultaneously. Similarly, analyzing the *in situ* bacterial compositions with metagenomic approaches has been widely used to interpret the role of bacteria in CRC.^1,2^ However, these methods fail to elucidate the functional implications of the microbiome activity in the TME. Instead, transcriptomics offers a way to examine a cell’s physiological state in a specific environment. Conventional methods restricted the analysis to mRNA changes in the bacteria or the host cell, limiting the understanding of host-microbiota interactions from the same samples. However, advances in RNA sequencing now enable “dual-RNAseq” studies, simultaneously capturing transcripts from pathogens and the host.^16^ Recently, metatranscriptomic studies of both the host and bacteria (dual-RNAseq) have investigated the bacteria and host interplay in some infectious diseases,^17,18^ but only few metatranscriptomic studies in CRC exist.^19,20^

In this study, we characterize the interplay between the host and active mucosa-associated bacteria in CRC, paired normal, and healthy tissue using fluorescence *in situ* hybridization and dual-RNAseq. Here, we show that biofilms and CRC core pathogens alter the TME, fueling an inflammatory response with pro-tumorigenic potential and a shift in the immune phenotype. *Fusobacterium spp*. was associated with accumulating bacterial biomass (biofilms) and acute inflammation in CRC samples. These observations were supported by RNAseq, showing that samples with high bacterial activity displayed a pro-inflammatory profile. Notably, overall bacterial activity seemed more critical for the immune phenotype, affecting various immune cells, including M2 macrophages, regulatory T-cells, and myeloid dendritic cells. These findings emphasize the complexity of the relationship between gut microbiota and CRC pathogenesis and the importance of widening the scope beyond core pathogens when investigating the functional implications of microbiota activity in the TME.

## Materials and methods

### Patient recruitment and ethics

Patients were recruited at Zealand University Hospital from December 2020 to December 2021. Inclusion criteria were persons >18 years old with written, approved consent admitted for a colonoscopy exam or patients undergoing surgery. Subjects were divided into patients with pathologically verified CRC (any T-stage) and healthy persons with no underlying gastrointestinal malignancies or diseases. This study was approved by the Danish Regional Ethical Committee (SJ-826) and the Danish Data Protection Agency (REG-024-2020).

### Study design and material

Colorectal cancer RNA sequencing data from The Cancer Genome Atlas https://portal.gdc.cancer.gov was used to estimate the required sample sizes. Based on the bacterial gene transcription, it was estimated that 40 patients should be included in each group. An alpha of 0.5 and a power of 0.8 was used. Mucosal colon biopsies were collected from patients with pathologically verified CRC (any T-stage). Biopsies were collected from the tumor and paired normal tissue >10 cm away from the tumor, if possible. Mucosal colon biopsies were also collected from individuals with no observed gastrointestinal diseases as healthy control samples. A pathologist screened CRC biopsies to ensure the presence of carcinoma. After collection, biopsies for RNA sequencing were placed immediately in RNAlater® (Invitrogen, MA, USA). For microscopy analysis, two biopsies were collected per subjects in the groups. Biopsies were fixated immediately in 4% buffered paraformaldehyde (pH 7.4) and stored at 4°C for at least 24 hours before being embedded in paraffin (FFPE).

### Peptide nucleic acid (PNA) probes

PNA probes targetting *B. fragilis* (Bfrag-998)^21^ and *Fusobacterium spp*. (FUS714)^22^ were ordered from Biomers (Ulm, DE). Bfrag-998 was tagged at the 5‘end with Cyanine5 (Cy5–5´-GTTTCCACATCATTCCACTG-´3) and FUS714 was tagged at the 5‘end with Cyanine3 (Cy3–5’- GGCTTCCCCATCGGCATT-´3). A universal (BacUni) bacterial probe (AdvanDx, Woburn, MA) tagged at the 5‘end with Texas Red was used to visualize all bacteria. DAPI (4′,6-diamidino-2-phenylindole) was used as a DNA stain to visualize nuclei (Life Technologies, OR, USA). A detailed description of the staining protocol (fluorescence *in situ* hybridization) is described in Supplemental Material and Method.

### Bacterial biomass

One biopsy was selected for analysis in the different groups. Area (tile scan) and depth (z-stack) were manually set for each biopsy. A 594 nm laser was used to excite the BacUni probe, and the fluorescence emission was detected in 597–661 nm intervals. Biomass (µm^3^) was measured as previously described,^23^ using the Measurement Pro addon in Imaris 9.7.2 software (Oxford Instruments, UK). Imaris utilizes a pixel quantitative approach to measure biomass, where a mask was created for total biomass (tissue and bacteria) and bacterial biomass (only bacteria) according to the thresholding of fluorescence intensity.

### Probe validation

The specificity of Bfrag-998 and FUS714 was checked with the Basic Local Alignment Search Tool (BLAST) function in the NCBI database. The FUS714 probe aligned with other *Fusobacterium* strains, including four subspecies of *Fusobacterium nucleatum* (*polymorphum*, *nucleatum*, *vincentii*, and *animalis*), and it was also complementary to two other bacterial species belonging to the Fusobacteriaceae family (*Ilyobacter polytropus* and *Propionigenium modestum*); thus microscopy findings are referred to as *Fusobacterium spp*. The Bfrag-998 probe was specific for *B. fragilis*. Further, the FUS714 and Bfrag-998 probes were qualitatively validated to ensure correct differentiation during microscopy. The differentiation of *Fusobacterium nucleatum ssp. nucleatum* (ATCC 25,586) and *Bacteroides fragilis* (ATCC 25,285) were tested on spiked lung tissue explanted from a mink (surplus material from animal studies). Overnight cultures with both strains were prepared and grown in brain-heart infusion media (Sigma-Aldrich, USA) under anoxic conditions for 24 hours at 37°C. Tissue was spiked in a 1:1 ratio by injection. Afterwards, the tissue was fixated in 4% buffered paraformaldehyde, paraffin-embedded, and treated according to the PNA-FISH method described in Supplemental Material and Method. After initial testing and adjustment on spiked tissue, the probes were tested *ex vivo* on tumor biopsies with similar settings to ensure the correct differentiation of bacterial populations.

### *B.* *fragilis* and *Fusobacterium spp.* prevalence

Two sections with two biopsies were screened per group to assess the prevalence of *B. fragilis* and *Fusobacterium spp*. Excitation of DAPI was acquired at 405 nm, and the fluorescence emission was detected in 415–488 nm intervals. A 561 and 633 nm laser was used for the excitation of Cy3 (FUS714) and Cy5 (Bfrag-998), respectively. Fluorescence emission was detected in 549–573 nm intervals for FUS714 and 632–705 nm intervals for Bfrag-998. Sequential multiple-channel fluorescence scanning was used to avoid or reduce bleed-through (cross talk) across the fluorophores. Scattered cells or aggregated bacteria were counted and included in the prevalence measurement.

### Microscopy and image processing

All fluorescence microscopy was performed on an inverted Zeiss LSM 880 confocal microscope (Zeiss, Jena, DE), using either a Plan-Apochromat 63×/1.40 Oil DIC M27 objective or an EC Plan-Neofluar 40×/1.30 Oil DIC M27 objective. All images were obtained in 16-bit, and the instrument automatically assigned the optimal pinhole size (Airy units) for each analysis used. Illustrative images were processed with maximum intensity projection and a Gaussian filter for smoothing, and the brightness was increased by 20% for presentation purposes. All images were processed in the Imaris 9.7.2 software (Oxford Instruments, UK), either as 3D projections (bacterial biomass) or 2D projections (illustrative images). In addition, pseudo-coloring was used for representative images to separate different bacterial populations from each other. *Fusobacterium spp*. were pseudo-colored green, *B. fragilis* red, and all other bacteria purple.

### Histopathology and inflammation score

Two pathologists evaluated the histopathology and scored inflammation in CRC samples in a blinded way. Tissue sections were mounted on glass slides and stained with Hematoxylin & Eosin. A score was given for acute and chronic inflammation, reflecting the infiltration of polymorphonuclear leukocytes (PMNs) and lymphocytes, respectively. The degree of inflammation was scored 0 (no inflammation), 1 (mild inflammation), 2 (moderate inflammation), and 3 (severe inflammation).^24,25^

### Bacterial community composition

RNA extraction, rRNA depletion, library preparation, and preliminary analysis are described in detail in Supplemental Material and Method. Trimmed reads were classified using Kraken v.2.1.2^26^ to determine bacterial community composition using the standard RefSeq index database (obtained from: https://benlangmead.github.io/aws-indexes/k2). Abundances of actively transcribing bacteria were estimated with Bracken v2.7.^27^ The bracken output was multiplied by a scaling factor to account for differences in sequencing depth between sequencing runs. This scaling factor was calculated as the number of reads in the sample with the lowest number divided by the number of reads in a given sample. Scaled counts were used when comparing across the samples, and unscaled counts were used when comparing within-sample variation. Also, a threshold was applied to remove noise from low-abundance taxa. The cutoff was determined by visual inspection of the log-10 transformed scaled counts distribution, which was bimodal (Figure S1), and the intersection between the two independent, overlapping, normal-distributed populations was used as the cutoff (0.9).

### Differential gene expression and functional enrichment analysis

Trimmed reads were aligned to the human reference genome, and reads mapping to annotated gene-level features were quantified. Count data were filtered to include only protein-coding transcripts using the biomaRt package in R.^28^ Cancer status (“CRC”, “no_CRC”) was encoded as a dummy variable, cancer. Differential gene expression analysis was performed using the DEseq2 v1.36.0 with the design “~ cancer”. Differentially expressed genes (DEGs) with an adjusted p-value <0.05 and |log2 fold-change|>2 were used for pathway enrichment using a previously published approach.^29^ Pathways with a minimum of 25 and maximum of 85 genes and overlap of at least five genes with the DEGs of interest were included. Fisher’s exact test was performed for each pathway, and the resulting p-values were adjusted using the Benjamini-Hochberg correction. This analysis was performed separately for genes showing positive or negative log2 fold changes. To analyze the effect of bacteria on CRC tissue, the gene-count matrix was further subsetted to include only CRC samples. A binary variable was assigned to samples containing a high or low bacterial signal. Samples with high bacterial signals were defined as outliers (>1.5 times the interquartile range) on a normal distribution of scaled count (Figure S1). Differential gene expression and functional enrichment analysis were then repeated with this variable. In addition, *B. fragilis* and *F. nucleatum* specific genes were determined in CRC samples to investigate which genes were expressed specifically in the TME. A detailed description of the method can be found in Supplemental Material and Method.

### Immune cell profiling

There are many different methods for estimating immune cell infiltration from RNA sequencing data.^30,31^ Each of these likely returns different scores due to the underlying algorithm employed by the method and its predefined immune cell populations. Therefore, a consensus approach was utilized using the R package immunedeconv.^32^ This package implements multiple methods for estimating sample immune infiltration, including quantiseq, epic, estimate, mcp_counter, xcell, consensus_tme, and timer. Filtering and normalization of the raw-count matrix of gene expression values were performed with limma.^33^ Low-expressed genes were filtered out with the filterByExpr function, the scaling factor was estimated with calcNormFactors, and a matrix of TMM (Trimmed Mean of M-values) counts was generated with the cpm function. Generalised cell categories were defined for each cell type to account for different sets/subsets of immune cell types in the methods, e.g., CD4+ and CD8+, and regulatory T-cells were classified as T-cells. Also, given that quantiseq and epic return proportions, while mcp_counter, xcell, consensus_tme, timer, and estimate return scores on varying scales, the analyses were performed separately for proportions and scores. Further, normalization of the scores was performed by positive centering to adjust for differences in scales between scoring methods, adding the smallest observed score value (greater than zero) in each method as a pseudo count to scores that were 0, after which the values were log2 transformed. Finally, heatmaps were generated to visualize the hierarchical clustering of samples according to the immune cell profile score. The R-packages NBclust, cluster, and the results from the hierarchical clustering were then used to select the optimal number of immune cell clusters (*n* = 4). A nonparametric Kruskal-Wallace test was used to test whether the total bacterial, *F. nucleatum*, or *B. fragilis* activity differed between the immune cell clusters. Post-hoc, pairwise comparisons between clusters were then performed with a Dunn test to test which clusters had a specifically higher or lower activity. Finally, a separate analysis was performed using ordinary least squares regression to assess how total bacterial, *F. nucleatum*, or *B. fragilis* activity affected the immune phenotype across sample types (CRC, paired normal, and healthy tissue). The model was fit to the immune score for a single immune cell type (response variable) with sample type and log-10 scaled counts as responses. This was repeated for all immune cell types across all deconvolution methods.

### Statistics

Bacterial biomass (µm^3^) was log-10 transformed (Log) to ensure normally distributed data unless otherwise stated. In some cases, CRC data were separated into high and low bacterial biomass using the average of all bacterial biomass measurements as the cutoff (4.2 log10(um^3^). Samples with the missing paired were excluded from all paired analyses. Graphs and statistics were carried out with either GraphPad Prism 9.3.1 (GraphPad Software, La Jolla, California, USA) or the R software v3.6.0 (R Development Core Team 2004). An adjusted p-value was reported in the case of multiple testing, and a p-value ≤ 0.05 was considered significant.

## Results

### Patient characteristics

Standard osmotic bowel prep was used in all cases before sampling, and only two patients with CRC reported using antibiotics before inclusion and sampling. Patients with CRC had a higher mean age and ASA score than healthy persons, more males were diagnosed with CRC, and left-sided tumors were more prevalent (Table 1). These differences were expected, given the etiology of CRC and the demographic characteristics of the CRC population. Ten patients did not receive pathologically verified tumor staging due to advanced disease; palliative care was provided in these cases rather than surgery.

**Table 1.** Population characteristics.

Characteristics	CRC, *N* = 40	Healthy, *N* = 40	P-value
Sex, male (%)	24 (60.0)	25 (62.5)	>0.99
Age, median (range)	76.5 (47–90)	68.5 (46–86)	0.03
Weight in Kg., median (range)	73.5 (46–119)	76 (49–121)	0.58
BMI, median (range)	24.6 (16.7–41.2)	25.8 (17.6–42.9)	0.33
Missing information (BMI)	2	3	
Anatomic location of sampling (%)			
Left-sided	29 (72.5)	22 (55.0)	0.16
Right-sided	11 (27.5)	17 (42.5)	
Missing information (anatomic location)		1	
T-stage (%)			
1	4 (10.0)	N/A	N/A
2	7 (17.5)	N/A	N/A
3	14 (35.0)	N/A	N/A
4	5 (12.5)	N/A	N/A
Missing information (T-stage)	10		
Lymph node metastases, N (%)	13 (32.5)	N/A	N/A
Distant metastases, M (%)	8 (20.0)	N/A	N/A
Missing information (N/M)	3/2		
pMMR/dMMR	36/2	N/A	N/A
ASA score (%)			<0.01
1	2 (5.0)	6 (15.0)	
2	24 (60.0)	20 (50.0)	
3	14 (35.0)	1 (3.7)	
Missing information (ASA score)		13	
Smoking (%)			
Yes	10 (25.0)	3 (7.5)	0.33*
No	12 (30.0)	14 (35.0)	
Previously	17 (42.5)	15 (37.5)	
Missing information (smoking)	1	8	
Diabetes (%)			0.41
DM1	1 (2.5)	0 (0.0)	
DM2	10 (25.0)	6 (15.0)	
Missing information (diabetes)		6	

ASA, American Society of Anesthesiologists. BMI, Body Mass Index (Kg/m^2). DM, Diabetes mellitus. N/A, not applicable. *Current and previous smoking and DM1 and DM2 have been pooled for statistical analysis. Continuous data was tested with a two-sided student t-test or Mann-Whitney Test, and categorical data was tested with a chi-square test. A p-value ≤ 0.05 was considered statistically significant.

### Increased bacterial biomass was observed in CRC tissue

**Figure 1.**
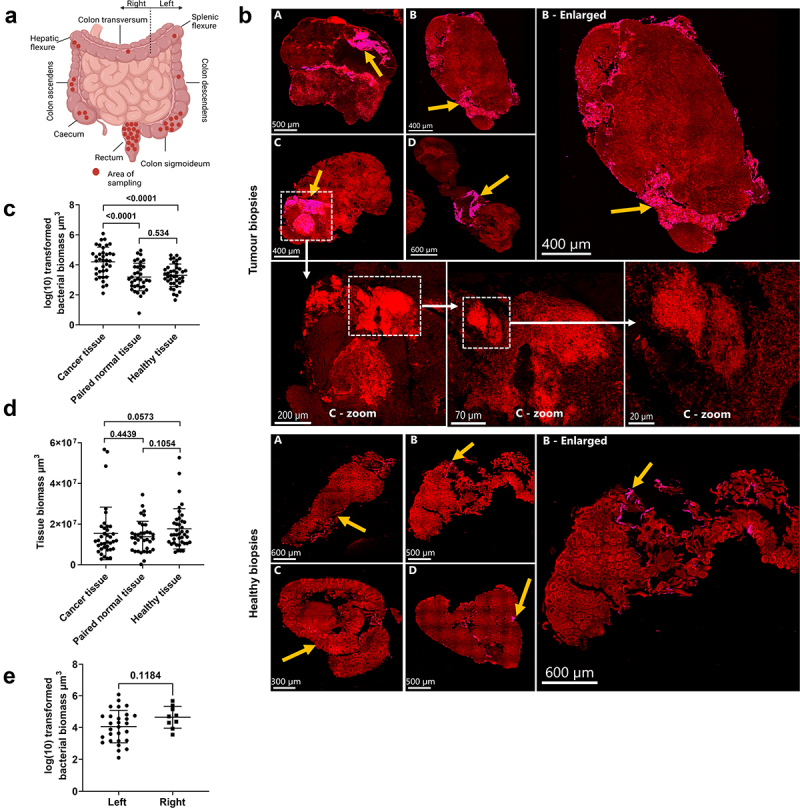
Assessment of bacterial biomass in biopsies collected from patients with and without CRC. (a) Diagram showing the anatomical sampling of CRC biopsies. (b) Cross-sections of tumour biopsies and healthy tissue biopsies showing the distribution of bacterial biomass. Yellow arrows indicate the area with bacterial biomass. Dashed boxes and white arrows show the morphological evidence of bacteria in the cross-section of a tumour biopsy (image C). Tissue was visible via autofluorescence. Scale bars are shown in the lower-left corner of all images. (c) Logarithmic (log) transformed bacterial biomass measured in cubic micrometres (µm^3^) from collected biopsies. (d) Total tissue biomass (bacteria and tissue) measured in µm^3^ from collected biopsies. (e) Log-transformed bacterial biomass on the left- and right-sided tumour biopsies measured in µm^3^. statistical comparison was carried out with paired and unpaired t-tests (C), paired t-test and Mann-Whitney test (D), and unpaired t-test (E). Bars represent standard deviation (SD). A p-value ≤0.05 was considered statistically significant.

Bacterial biomass (µm^3^) was quantified systematically in cross-sectioned whole biopsies using pan-microbial PNA-FISH. Three pairs of samples (CRC and paired normal tissue) were excluded due to non-cancerous origin (*n* = 2) and incorrect processing (*n* = 1). In one case, it was not possible to sample paired normal tissue. No differences were observed between groups due to the removal of samples (Table S1). Biopsies were mainly collected from the rectum and colon sigmoideum (Figure 1(a)). The bacterial biomass displayed a tissue-invasive phenotype in CRC biopsies, while bacteria were localized along the epithelial lining of healthy colon biopsies (Figure 1(b)). Large patches of aggregated bacteria (biofilm) were frequently observed in CRC tissue, and bacterial biomass was 11 to 17-fold higher in CRC tissue compared to paired normal and healthy tissue (Figure 1(c)). There were no differences in biopsy sizes across the groups (Figure 1(d)). When stratifying the bacterial biomass into the respective anatomic locations of sampling, a stepwise increase in mean (log(10)) bacterial biomass was observed from the rectum (3.93 ± 0.90 SD) over sigmoideum (4.32 ± 1.01 SD) to colon ascendens (4.35 ± 0.65 SD) and cecum (4.76 ± 0.72 SD); however, this was not significant. Similarly, no differences (mean difference = 0.59 ± 0.37 SEM, *p* = 0.12) were observed when stratifying into left- and right-sided tumors (Figure 1(e)), as previously reported.^34,35^ Bacterial biomass was not associated with tumor staging (T1-T4), lymph node metastasis (N), or distant metastasis (M) (Figure S2).

The heterogeneous distribution of biofilms across the whole section (Figure 1(b)) highlights the importance of measuring bacterial biomass across whole biopsies and suggests that bacterial effects on the TME might be local, potentially even within individual biopsies.

### The prevalence of *Fusobacterium spp*. correlated with increased bacterial biomass and virulence expression profile in CRC tissue

**Figure 2.**
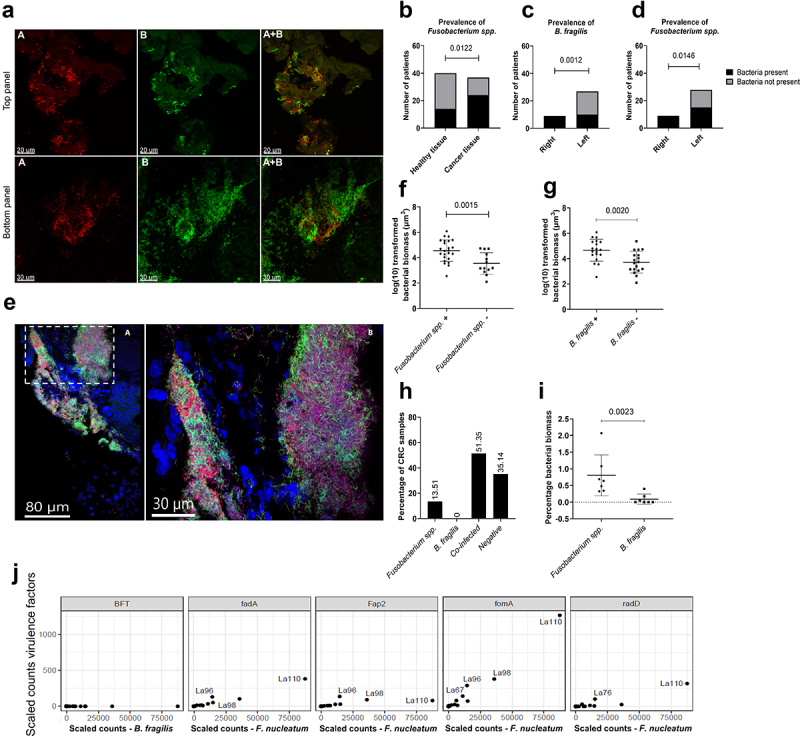
Prevalence of *Fusobacterium spp*. and *B. fragilis* in CRC, paired normal, and healthy tissue. (a) Representative images showing the qualitative separation of FUS714 (*Fusobacterium spp*.) from bfrag-998 (*B. fragilis*) in spiked tissue (top panel) and tumour tissue (bottom panel). (b) Prevalence of *Fusobacterium spp*. in healthy tissue and cancer tissue. (c,d) Prevalence of *B. fragilis* (C) and *Fusobacterium spp*. (d) in left- and right-sided tumours. (e) Representative images showing mixed-species biofilms with *B. fragilis* (red), *Fusobacterium spp*. (green), other bacteria (purple), and host cells (blue). Image A is an overview image, and the white dashed box shows the enlarged area in image B. (f,g) correlation between the prevalence of *Fusobacterium spp*. (B) and *B. fragilis* (C) and logarithmic (log) transformed bacterial biomass in CRC tissue measured in cubic micrometres (µm^3^). (h) Percentage of CRC samples positive with either *Fusobacterium spp*., *B. fragilis*, co-infected or negative. (I) The percentage of bacterial biomass for *B. fragilis* and *Fusobacterium spp*. on a subset of co-infected samples (*n* = 7) with high bacterial biomass. (J) Scaled counts assigned to the *B. fragilis* toxin (BFT) and *F. nucleatum* virulence factors FadA, Fap2, FomA, and RadD. Scale bars are shown in the lower-left corner of all images. Statistical comparison was carried out with Fisher’s exact tests (B+C+D), unpaired t-tests (F+G), and Mann-Whitney tests (I). Bars represent standard deviation (SD). A p-value ≤ .05 was considered statistically significant.

Species-specific PNA-FISH was used to assess the prevalence and contribution of *Fusobacterium spp*. and *B. fragilis* to bacterial biomass in CRC. Successive separation of the probes was initially tested in spiked tissue and tumour tissue (Figure 2(a)). *Fusobacterium spp*. were observed in 24 out of 37 (64.9%) tumour biopsies, 18 out of 36 (50.0%) paired normal biopsies, and 14 out of 40 (35.0%) healthy biopsies. *B. fragilis* was observed in 19 out of 37 tumour biopsies (51.4%), 15 out of 36 paired normal biopsies (41.7%), and 13 out of 40 healthy biopsies (32.5%). The prevalence of *Fusobacterium spp*. was significantly higher in CRC tissue than in healthy tissue (Figure 2(b)). Interestingly, enrichment was observed for both bacteria in right-sided tumors (Figures 2(c,d)), suggesting anatomical preference. No correlation between prevalence and tumor staging (T1-T4), lymph node metastasis (N), or distant metastasis (M) was observed (Figure S2).

Microscopy revealed that *Fusobacterium spp*. formed a substantial proportion of the mixed-species biofilms in CRC tissue (representative image in Figure 2(e)), suggesting superior host-cell adhesion or facilitated co-adhesion of other bacteria. A sub-group analysis revealed that samples positive with *Fusobacterium spp*. had a higher mean percentage of bacterial biomass (1.1% vs. 0.1%) than those without *Fusobacterium spp*. (Figure 2(f)). Similarly, the mean bacterial biomass in samples with *B. fragilis* was higher than those without (Figure 2(g)); however, we hypothesized these findings were confounded due to co-infection by *Fusobacterium spp*. (Figure 2(h)). Therefore, the bacterial biomass of *Fusobacterium spp*. and *B. fragilis* was analyzed in seven co-infected samples with high bacterial biomass. *Fusobacterium spp*. was more abundant than *B. fragilis* in these samples, and a difference was observed in the mean percentage of bacterial biomass (Figure 2(i)). Moreover, the expression patterns of *B. fragilis* enterotoxin (BFT) and different cell and bacteria-adhesive virulence factors of *F. nucleatum* were analyzed. Tissue and co-aggregating properties are well described for *F. nucleatum*.^36–39^ Accordingly, the virulence factors FadA, Fap2, FomA, and RadD were highly expressed in samples with high *F. nucleatum* counts (Figure 2(j)), suggesting a putative role in the buildup of bacterial biomass. Surprisingly, in contrast to the high prevalence of *B. fragilis* in CRC samples, no expression of BFT was observed. *B. fragilis* toxins are more common in right-sided tumors,^40^ and the inclusion of a few right-sided tumor biopsies in this study might have influenced the findings.

### Bacterial biomass was associated with acute inflammation and co-localised with necrotic areas in CRC tissue

**Figure 3.**
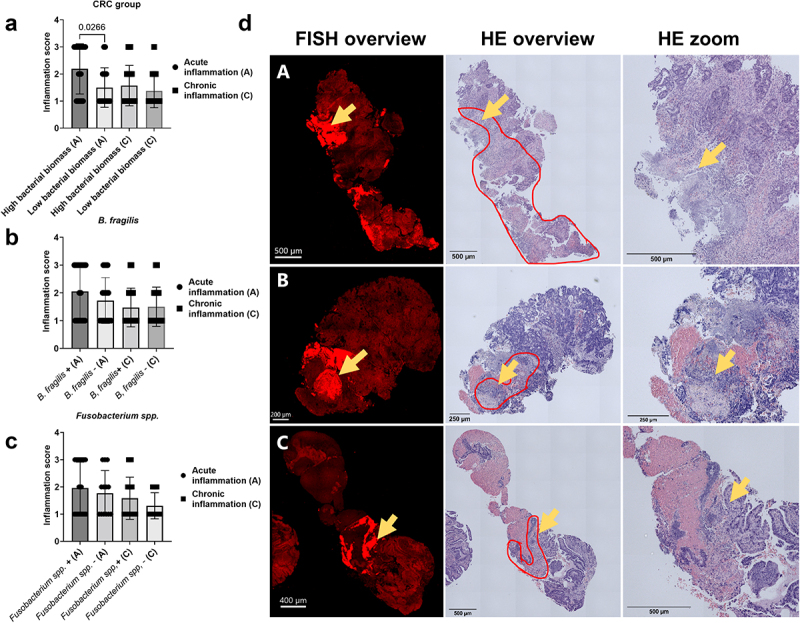
Histopathological evaluation of the bacterial influence on the TME. (a) Inflammation score for CRC samples with high and low bacterial biomass, divided into acute and chronic inflammation. (b,c) Inflammation score for CRC samples with (+) and without (-) *B. fragilis* (B) and *Fusobacterium spp*. (C), divided into acute and chronic inflammation. (d) Representative images showing the co-localisation of bacterial biomass and necrotic tissue on cross-sectioned tumour biopsies. The red encircled area indicates the area with necrosis, and the yellow arrows indicate the area with bacterial biomass. All biomass measurements were performed with the Imaris software through thresholding of fluorescence intensity. Tissue was visible via autofluorescence. Scale bars are shown in the lower-left corner of all images. Statistical comparison was carried out with Mann-Whitney tests (A+B+C). Bars represent standard deviation (SD). A p-value ≤ 0.05 was considered statistically significant.

Inflammation scores, reflecting PMNs and lymphocyte infiltration, were given for acute and chronic inflammation to determine if bacteria influenced the TME. Samples with high bacterial biomass had a higher degree of acute inflammation than samples with low bacterial biomass (Figure 3(a)). Similarly, there was a higher degree of inflammation in samples with *B. fragilis* and *Fusobacterium spp*.; however, this was not significant (Figures 3(b,c)). No inflammation was observed in healthy biopsies (data not shown). Moreover, in 18 out of 21 samples (85.7%) with high bacterial biomass, it was observed that bacteria and necrotic tissue were co-localized (representative images shown in Figure 3(d)), suggesting an anatomical preference for biofilm growth or that bacterial metabolites or products are involved in the malignant transformation.

### Higher counts for bacteria, *F.* *nucleatum*, and *B.* *fragilis* were observed in CRC tissue

Dual-RNAseq was performed on 118 samples to assess the bacterial activity within CRC, paired normal, and healthy tissue. One healthy sample was omitted because of a failed library preparation, and tissue collection was unattainable for one paired normal sample. Reads were taxonomically assigned, classified, scaled, and quantified for each sample (Figure S1). Sample type described the majority of sample variation (Figure S3).

**Figure 4.**
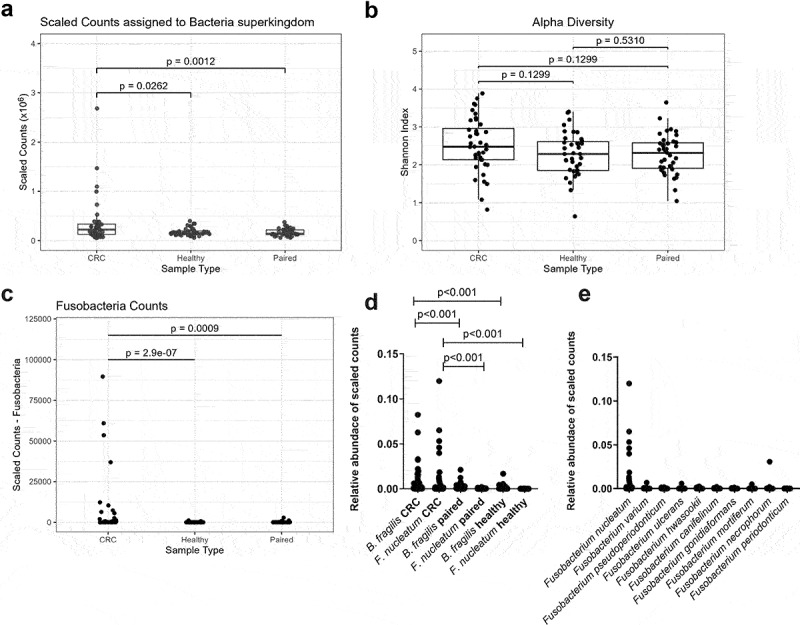
Characterisation of bacterial richness and diversity in CRC, paired normal, and healthy tissue. (a) Scaled counts assigned to bacteria in CRC, healthy, and paired normal tissue. (b) The alpha diversity in CRC, healthy, and paired normal tissue was compared with the Shannon index. (c) Scaled counts assigned to Fusobacteria in CRC, healthy, and paired normal tissue. (d) Relative abundance of scaled counts assigned to *B. fragilis* and *F. nucleatum* in CRC, paired normal, and healthy tissue. (e) Relative abundance of scaled counts assigned to *Fusobacterium spp*. in CRC tissue. Statistical comparison was carried out with Wilcoxon signed-rank test and Wilcoxon rank-sum-test (A+C+D), paired and unpaired t-tests (B), and Mann-Whitney tests (D). Bars represent standard deviation (SD). A p-value ≤ 0.05 was considered statistically significant.

Bacterial counts were higher in CRC tissue (Figure 4(a)) than in paired normal or healthy tissue; however, the groups did not differ in alpha diversity (Figure 4(b)). In general, Firmicutes and Proteobacteria were the dominant phyla across all groups (Figure S4A-S4C), while an increase of Bacteroidota, Fusobacteria, and Actinobacteria was mainly observed in the CRC group (Figure S4D – S4(f)). Similar to previous findings, Fusobacteria (Figure 4C) counts were higher in CRC tissue than in healthy or paired normal tissue, and *F. nucleatum* and *B. fragilis* counts were higher in CRC tissue (Figure 4(d)) than in healthy or paired normal tissue. *F. nucleatum* comprised 78.9% of the total counts assigned to the Fusobacteria phylum. Other *Fusobacterium spp*. were present in CRC samples; however, *F. nucleatum* was most abundant (Figure 4(e)). These results confirms the enrichment of the two core pathogens in CRC tissue and suggest that increased findings of *Fusobacterium ssp*. in the samples subjected to microscopy probably were due to the presence of *F. nucleatum*.

### Higher bacterial activity in CRC tissue affects host transcription

**Figure 5.**
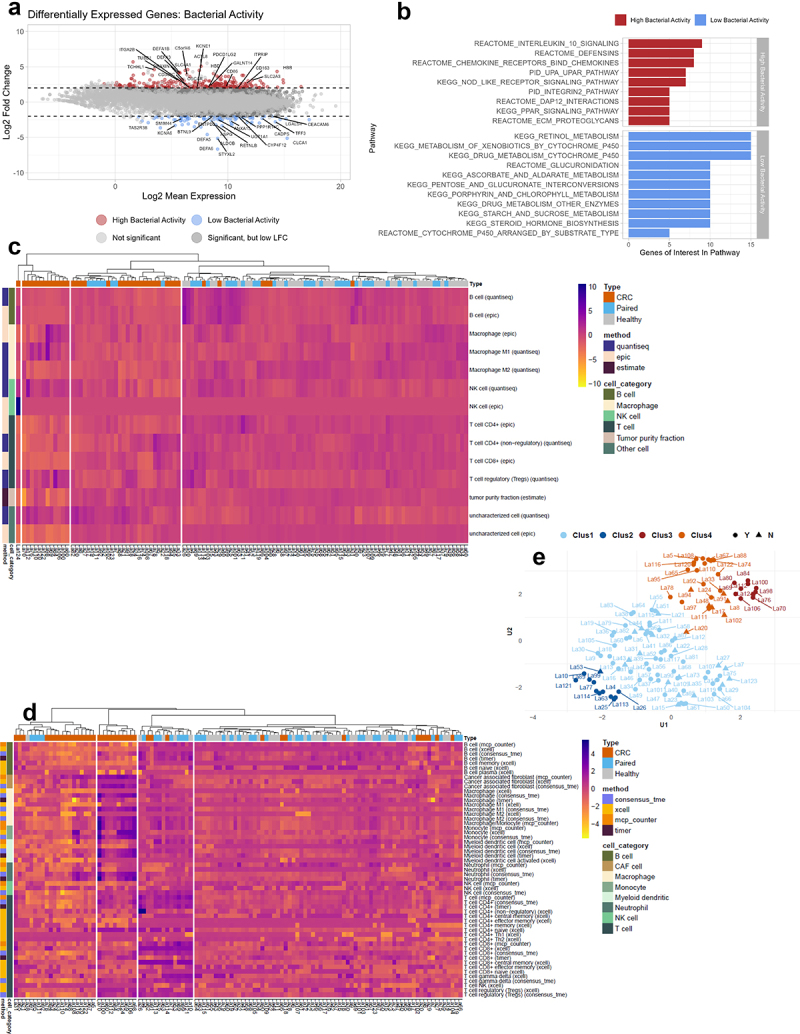
Differentially expressed genes (DEGs) and tissue immune phenotype. (a) MA plot showing the distribution of significantly differentially expressed genes between CRC tissue with high and low bacterial activity. Colouring highlights the 20 most significant DEGs with an adjusted p-value less than 0.05 and absolute log2 fold-change >2. (b) The Kyoto Encyclopedia of genes and genomes (KEGG), pathway interaction database (PID), and REACTOME (a database of reactions, pathways, and biological processes) databases were used to identify pathways that were significantly enriched or decreased in CRC tissue with high and low bacterial activity. (c,d) Heatmaps showing immune cell profiles in CRC, healthy, and paired normal tissue, presented as fractions from the quantisec, epic, and estimate immune scoring systems (C) and normalized scores from the concensus_tme, xcell, mcp_counter, and timer immune scoring systems (D). Colouring from yellow (−4) to purple (4) indicates the degree of infiltration, where purple is high infiltration. (e) Four immune cell profile clusters (Clus1-Clus4) were defined in this study based on the hierarchical clustering of samples according to immune cell infiltration in the heatmaps. Y and N indicate whether samples were stable to the assigned clusters.

To assess the effect of increased bacterial activity on the TME, differential gene expression and functional enrichment analyses were performed between the CRC samples containing high (*n* = 6) and low (*n* = 34) bacterial RNA. This analysis identified 332 DEGs due to bacterial activity in CRC, where 252 showed increased expression with increased bacterial activity (Figure 5(a)). These included increased expression of several proinflammatory cytokines (*CXCL6*, *CXCL8*, *CXCL9*, *IL1B*, *IL6*, *CCL3*, and *CCL7*), defensins (*DEFA1*, *DEFA3*, *DEFA4*, *DEFB125*, *DEFB129*, and *DEFB131*), matrix-metalloproteases (*MMP1*, *MMP12*, and *MMP13*) and other immunomodulatory factors and receptors (*S100A8*, *MEFV*, *CD86*, *CSF3*, *FPR1*, *PTGS2*, and *TLR*). These genes represented significant enrichment of IL-10 signaling, defensin, chemokine, and other pathways (Figure 5(b)). Further, metabolic pathways involving UDP-glucuronosyltransferases (*UGT1A1, UGT1A10, UGT1A4, UGT1A7, UGT1A8, UGT1A9*, *UGT2B15*, and *UGT2B17*), alcohol dehydrogenases (*ADH1B* and *ADH1C*), and cytochrome P450 genes (*CYP2B6, CYP2C18, CYP2C19, CYP2B6, CYP2C18, CYP2C19*, and *CYP4F12*) showed significantly increased expression in samples with low bacterial activity. Interestingly, many of these significantly enriched pathways in CRC samples with low bacterial activity (Figure 5(b)) overlapped with significantly enriched pathways in healthy samples compared with CRC samples (Figure S5). Next, all the reads that did not align with humans were aligned to *B. fragilis* and *F. nucleatum* (Figure S6) to investigate which genes were expressed specifically in the TME. Many up-regulated genes overlapped between the core pathogens (Table S2), and the genes seemed to be universal or at least shared by many species, meaning that a species-specific contribution was impossible to infer. Nevertheless, some potentially interesting genes were up-regulated (*tuf*, *gap*, and *dnak*), which have been implicated in deleterious processes, such as adhesion and invasion, immune system evasion, and disruption of DNA repair mechanisms.^35,41,42^ These results demonstrate that increased bacterial activity and potentially species-specific gene transcription detrimentally impact the TME by altering host gene transcription, manifesting a pro-inflammatory signature.

### Higher bacterial activity in CRC tissue affects the immune phenotype

We then investigated whether increased overall bacterial or species-specific activity affected the immune phenotype in CRC tissue. The analysis was conducted across immune cell clusters and sample types (CRC, paired normal, and healthy). Several existing cell deconvolution and immune scoring systems were integrated to develop a more robust estimate and stratified by whether the output was proportional (Figure 5(c)) or a normalized score (Figure 5(d)). This analysis included 115 samples (3 were identified as outliers and removed from the dataset). Samples were grouped into four clusters with different immune profiles (Figure 5(e)), which appeared to separate healthy and paired normal samples (Clusters 1 and 2) and CRC samples (Clusters 3 and 4). The overall bacterial and species-specific activity significantly differed between clusters (Table S3) but showed no associations with specific immune-cell subtype abundance herein (data not shown). However, when assessing the effect of overall and species-specific activity across sample types, increased bacterial activity correlated with the infiltration of macrophage/monocyte, myeloid dendritic cells, regulatory T-cells, tumor purity fraction, and tumor purity score in CRC tissue (Table 2). *F. nucleatum* activity did not correlate with an increased presence of immune cells, while *B. fragilis* correlated with the tumor purity fraction score and infiltration of neutrophils (Table 2). The analysis was also conducted excluding paired normal samples, as dysbiosis has been suggested to occur in the whole colon.^43^ Similar hierarchical sample clustering was observed (Figure S7). Bacterial and species-specific activity varied significantly between clusters (Table S3) but showed no associations with immune cells. When assessing across sample types, *F. nucleatum* activity correlated with infiltration of effector memory CD4+ T-cells (Table 2). In addition, several other immune scores were associated with increased total bacterial activity (Table 2), including the immune and microenvironment scores. An overview of all the immune cell subtypes and methods used can be found in Tables S4-S9.

**Table 2.** Bacterial groups impacting infiltration of specific immune cells in CRC tissue.

Specific counts	Immune cell group	Strength of association	Methods showing a correlation
**Paired normal and healthy tissue samples were used as controls**			
Bacteria	Macrophage/Monocyte	p < 0.01	1 out of 1
Bacteria	Myeloid dendritic cells (activated)	p < 0.05	1 out of 1
Bacteria	Regulatory T-cells	p < 0.001	1 out of 2
Bacteria	Tumour purity fraction	p < 0.001	1 out of 1
Bacteria	Tumour purity score	p < 0.05	1 out of 1
*B. fragilis*	Neutrophils	p < 0.05	1 out of 4
*B. fragilis*	Tumour purity fraction	p < 0.05	1 out of 1
**Healthy tissue samples were used as controls**			
Bacteria	Eosinophils	p < 0.05	1 out of 2
Bacteria	Hematopoietic stem cell	p < 0.01	1 out of 1
Bacteria	Immune score	p < 0.05 and *p* < 0.01	2 out of 3
Bacteria	Macrophage M2	p < 0.05 and *p* < 0.05	2 out of 2
Bacteria	Macrophage/Monocyte	p < 0.01	1 out of 1
Bacteria	Microenvironment score	p < 0.05	1 out of 1
Bacteria	Monocyte	p < 0.05 and *p* < 0.01	2 out of 3
Bacteria	Myeloid dendritic cells (activated)	p < 0.001	1 out of 1
Bacteria	Regulatory T-cells	p < 0.001	1 out of 2
Bacteria	Tumour purity fraction	p < 0.0001	1 out of 1
Bacteria	Tumour purity score	p < 0.01	1 out of 1
*F. nucleatum*	T cell CD4+ effector memory	p < 0.05	1 out of 1

These results demonstrate how bacteria and CRC core pathogens can affect the immune phenotype and suggest that including paired normal samples results in additional variability, which masks the interplay between bacteria and the host. Separating samples into immune clusters unveiled variations in species-specific counts between CRC and healthy clusters (Table S3). Notably, this distinction did not show species-specific effects on the prevalence of specific immune cell subtypes. This indicates that *B. fragilis* and *F. nucleatum* may have limited influence on the immune phenotype, particularly when assessing their influence across clusters with varying immune profiles.

## Discussion

The relationship between gut microbiota and CRC has been extensively studied, with multiple studies reporting an enrichment of core pathogens in the TME.^4–7^ *In vitro*, *in vivo*, and metagenomic investigations have provided valuable insights but are limited in elucidating the functional implications of microbiome activity within the TME. Therefore, we subjected mucosal biopsies to dual-RNAseq and FISH to explore how biofilm and the CRC core pathogens, *F. nucleatum* and *B. fragilis*, affected the TME. Our findings improve upon previous findings by showing that *Fusobacterium spp*. is associated with the accumulation of biofilms, fueling an acute inflammatory response. Additionally, our results showed that biofilms and bacterial activity impacted the transcriptional signature and immune phenotype more than individual core pathogens, emphasizing the importance of widening the bacterial scope beyond CRC core pathogens.

Microscopy findings in this study highlight the buildup of mixed-species biofilms in CRC samples, increased prevalence of *B. fragilis* and *F. nucleatum*, and a trend toward increased bacterial biomass in right-sided tumors, in agreement with previous studies.^34,40,44–46^ To our knowledge, *Fusobacterium spp*. has not previously been associated with increased bacterial biomass in CRC samples. This new observation, coupled with the degree of infiltrating PMNs and co-localization of biofilms with necrotic tissue in CRC samples, suggests a putative role for *Fusobacterium spp*. in the inflammatory response. *F. nucleatum* has previously been observed in ulcerated regions.^47^ Further, the intra-tumoral presence of other *Fusobacterium spp*. has previously been associated with an inflammatory response in CRC samples with a correlating expression of FadA,^48^ suggesting common pathogenic traits across *Fusobacterium spp*. The ability of *F. nucleatum* to adhere with other bacteria and tissues is well described.^36–39,49^ Accordingly, expression of the tissue-adhesive and aggregation-promoting virulence factors, FadA, Fap2, RadD, and FomA, was increased in samples with high *F. nucleatum* activity in this study, suggesting that *F. nucleatum* may facilitate the buildup of biofilms/bacterial biomass in CRC samples.

Samples with high bacterial activity showed increased signs of inflammation, while samples with low bacterial activity showed pathway enrichments resembling healthy samples. These findings highlight that increased bacterial activity negatively impacts the local TME by increasing the local inflammatory response. Samples with high bacterial activity exhibited a pro-inflammatory signature, including the expression of cytokines (IL6, CXCL8) and matrix-metalloproteases (MMP1), which correlates with findings from a study investigating the influence of high *F. nucleatum* presence in CRC samples.^50^ *CXCL8* codes for a chemoattractant that attracts neutrophils and other granulocytes (PMNs),^51^ suggesting an active host-microbiota interaction in the TME. In accordance with this, we observed an acute inflammatory response with PMN infiltration in samples with high bacterial biomass. Neutrophils and other PMNs can act protumorigenic,^52^ while IL-6 has been implicated in tumor growth and metastasis in CRC.^53^ Considering the infiltration of M2 macrophages and regulatory T-cells observed in samples displaying high bacterial activity in this study, alongside the association of *Fusobacterium spp*. with increased bacterial biomass, it is conceivable that *Fusobacterium ssp*. may indirectly drive a potent pro-tumorigenic inflammatory response. The effect of *F. nucleatum* has previously been evaluated according to consensus molecular subtyping (CMS) in CRC samples.^48,50,54^ While *F. nucleatum* did not show any association with the immune phenotype in one study,^54^ it was associated with a higher neutrophil infiltration, a higher abundance of regulatory T-cells and M2 macrophages and a low abundance of M1 macrophages in another study.^50^ Similarly, *Fusobacterium spp*. has been associated with an inflammatory response and the infiltration of granulocytes in the TME in patients with head and neck squamous cell carcinoma (HNSC),^55^ suggesting similar pathogenic traits across different cancers.

The species-specific association in our study included associations between *B. fragilis* and neutrophil infiltration and between *F. nucleatum* and effector memory CD4+ T-cell infiltration. Previous studies have evaluated the effect of *F. nucleatum* on CD4+ T-cell activity, with conflicting findings.^10,11,56^ To our knowledge, this is the first time that *B. fragilis* has been associated with neutrophil infiltration, though the high co-infection rate between *Fusobacterium spp*. and *B. fragilis* makes it difficult to infer a species-specific causation. Also, sub-dividing the dataset into high and low species-specific counts does not consider the role of other possible key pathogens. Thus, our findings should be interpreted as associations and further research is required to elucidate species-specific pathogenesis and mechanisms. We envision that conducting fecal transplantation experiments from patients with CRC to healthy mice populations could further verify the host-microbial interaction observed in this study, and subsequent *in* *vivo* studies with specific strains could elucidate mechanistic insights. Lastly, there seems to be a consensus that *F. nucleatum* enrichment is a hallmark for microsatellite instability (MSI) colorectal tumors and the CMS1 subgroup.^48,50,54,57^ Thus, including a few right-sided cancers and patients with dMMR may have affected the outcomes of this study regarding the influence of *F. nucleatum* on the TME.

Our results agree with a recent study by Zhao et al. describing the consensus mucosal microbiome from 924 CRC tumors.^3^ They included eight RNA datasets across different geographical locations and found the same phyla elevated in CRC tissue with no differences in alpha diversity, suggesting that our results are generalizable regarding bacterial composition. Further, our results build upon previous findings regarding the putative inflammatory role of the two core pathogens in CRC. However, our results also hold implications for future studies investigating the host-microbiota relationship. The function of *Fusobacterium spp*. to potentially serve as a foundation for subsequent colonization by other potentially pathogenic microorganisms highlights the importance of considering the community as a whole. Nevertheless, investigating the potential of species-specific targeting of *Fusobacterium spp*. or its virulence factors is an intriguing prospect for future clinical trials and novel therapeutic avenues. This approach could aim to reduce bacterial biomass and alleviate the accompanying inflammatory response. Bullman et al. showed that metronidazole treatment of patient-derived xenograft tumors with *F. nucleatum* reduced tumor growth.^47^ However, systemic use of antibiotics potentially fuels dysbiosis and oral administration has been associated with an increased risk of colon cancer.^58^ Targeting the virulence factors might address this concern, and a recent study suggested targeting FadA as a therapeutic opportunity.^48^ Alternatively, instead of targeting the bacteria, others have speculated about targeting host signaling pathways affected by *Fusobacteriales* to reduce the associated inflammation.^50^ In summary, upcoming clinical research is warranted to assess the safety of systemic or localized species-specific antibiotic treatments or interventions targeting virulence factors, alone or combined with approved anti-inflammatory therapies, to reduce bacterial biomass and the associated inflammatory response.

In conclusion, *Fusobacterium spp*. plays a role in the buildup of mixed-species biofilms, resulting in an increased inflammatory response. The influence of *B. fragilis* and *F. nucleatum* on the immune phenotype was modest in this study. In contrast, the collective presence of bacteria seemed more relevant in altering the immune phenotype and regulating genes and critical pathways. These observations confirm the narrative of the involvement of *F. nucleatum* and *B. fragilis* in CRC carcinogenesis while highlighting the importance of also widening the bacterial scope beyond CRC core pathogens when deciphering the role of bacteria in CRC carcinogenesis. These findings suggest a reduction in the bacterial biomass as a potential target to reduce inflammation-driven CRC carcinogenesis; however, there is a lack of clinical studies in this area, and future studies should explore the clinical implications of reducing bacterial biomass or targeting *Fusobacterium spp.*

## Supplementary Material

Supplemental Material

## Data Availability

The raw sequencing data and gene count tables produced in this study are not accessible to the public, as such an action would infringe upon patient consent and ethical regulations stipulated by the authorities in Denmark. Processed data devoid of sensitive information and original code supporting the results of this study is available in the Supplementary Information as well as in a Zenodo repository (DOI: https://zenodo.org/doi/10.5281/zenodo.10997467). Additional information is available upon reasonable request, and in the case of raw sequencing data, appropriate permissions are required for data accession.
