# Supplementary material for: Biofilms and core pathogens shape the tumor microenvironment and immune phenotype in colorectal cancer
Source: Gut Microbes. 2024 May 10;16(1):2350156. doi: 10.1080/19490976.2024.2350156 (PMC11093030; doi:10.1080/19490976.2024.2350156)
Supplement: Supplemental Material [file KGMI_A_2350156_SM7231.zip › Supplemental Figures 2.docx]

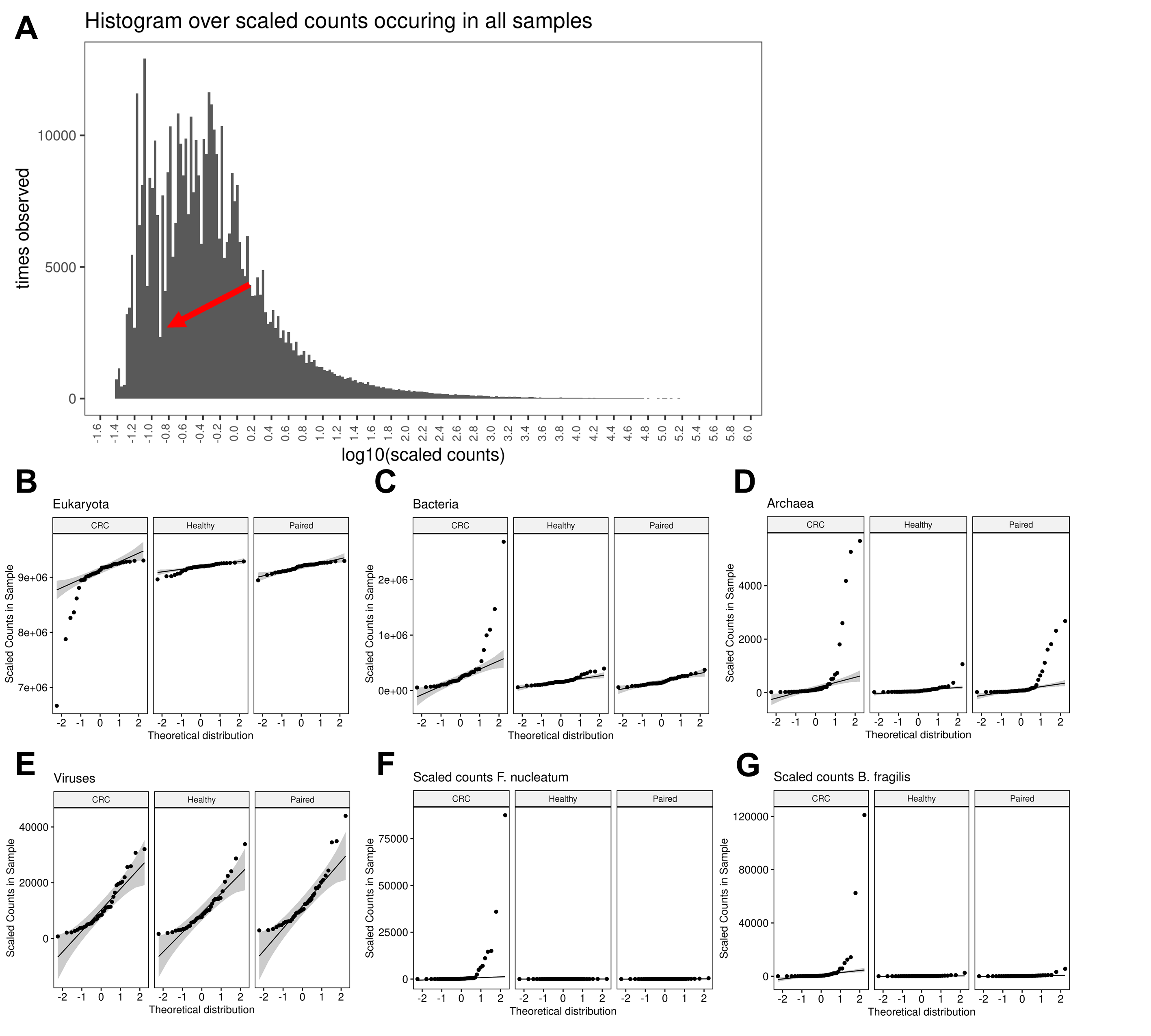


**Figure S1 - Noise filtering and distribution of counts assigned to Eukaryota, bacteria, archaea, viruses, *Fusobacterium nucleatum,* and *Bacteroides fragilis.***

**A**) Histogram showing the counts distribution over log-10 transformed scaled counts. The red arrow indicates the intersection between the populations, and all scaled counts < log-0.9 (indicated by red arrow) were set to 0 to remove noise. **B+C+D+E+F+G)** Normal distribution of scaled counts (y-axis) presented per sample across groups for Eukaryota (A), Bacteria (B), Archaea (C), virus (D), *F. nucleatum* (F), and *B. fragilis* (G).


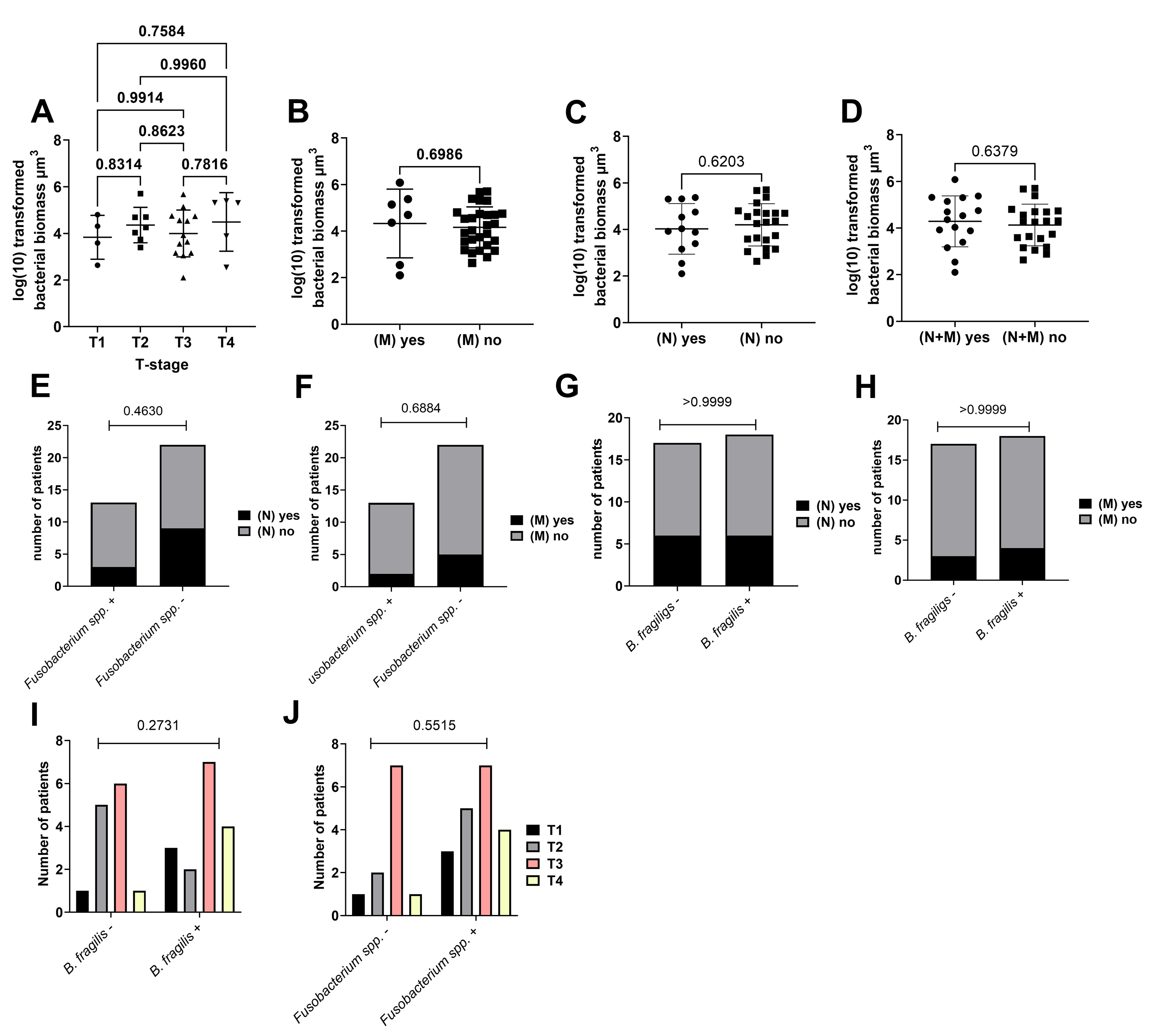


**Figure S2 – Bacterial biomass, Bacteroides fragilis, and Fusobacterium nucleatum prevalence was not associated with tumour staging, lymph node metastasis, or distant metastasis**

**A)** Logarithmic (log) transformed bacterial biomass measured in cubic micrometres (µm^3^) according to tumour stage (T1-4). **B+C+D)** Log-transformed bacterial biomass measured in µm^3^ according to distant (B) metastasis (M), Lymph node (C) metastasis (N), or both (D**).** All biomass measurements were measured with the Imaris software through thresholding of fluorescence intensity. **E+F)** Prevalence of Fusobacterium spp. compared with the number of patients with distant (M) metastasis or Lymph node (N) metastasis**. H+I)** Prevalence of Bacteroides fragilis compared with the number of patients with lymph node (N) metastasis or distant (M) metastasis**. J+K)** Tumor staging (T1-4) compared to the prevalence of B. fragilis (J) and Fusobacterium spp. (K). Statistical comparison was carried out with one-way ANOVA **(A),** unpaired t-test **(B+C+D)**, Fisher's exact t-test **(E+F+G+H),** and chi-square test **(I+J)**. Bars represent standard deviation (SD); a p-value ≤ 0.05 was considered statistically significant.

**
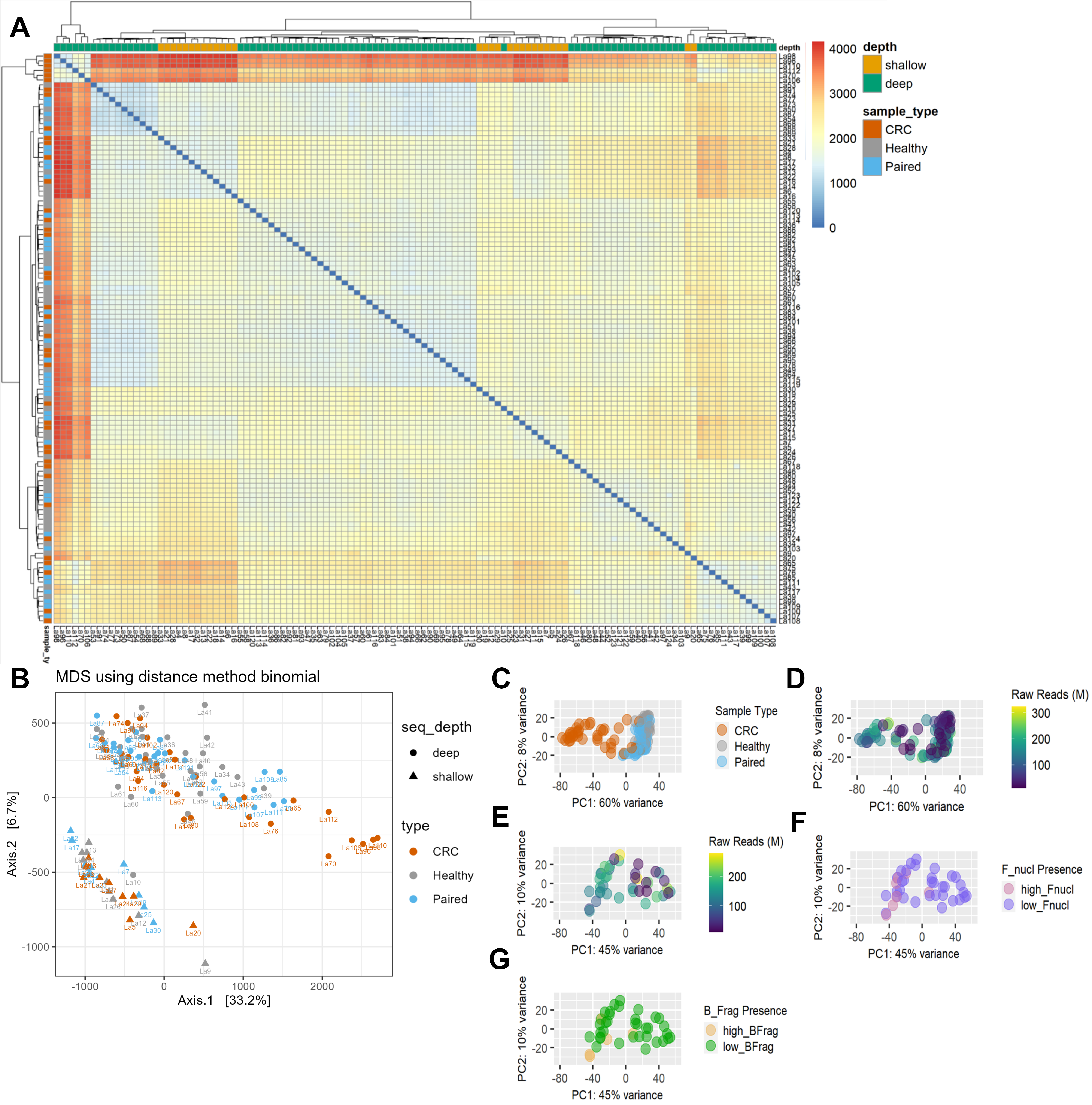
**

**Figure S3 - Principal-component analysis of normalized expression data.**

**A+B)** The clustering of CRC, healthy, and paired normal tissue samples according to sequencing depth (deep vs. shallow) is presented as a heatmap (A) and 2D scatterplot (B). **C)** All data is coloured according to groups. **D)** All data is coloured according to sequencing depth. **E)** CRC samples are coloured by sequencing depth. **F)** CRC samples are coloured according to the presence of *F. nucleatum*. Samples with high *F. nucleatum* counts were defined as those samples departing from the normal distribution in Figure S1, whereas low were those that followed the normal distribution. **G)** CRC samples are coloured according to the presence of *B. fragilis*. Samples with high *B. fragilis* counts were defined as those departing from the normal distribution in Figure S1, whereas low were those following the normal distribution.


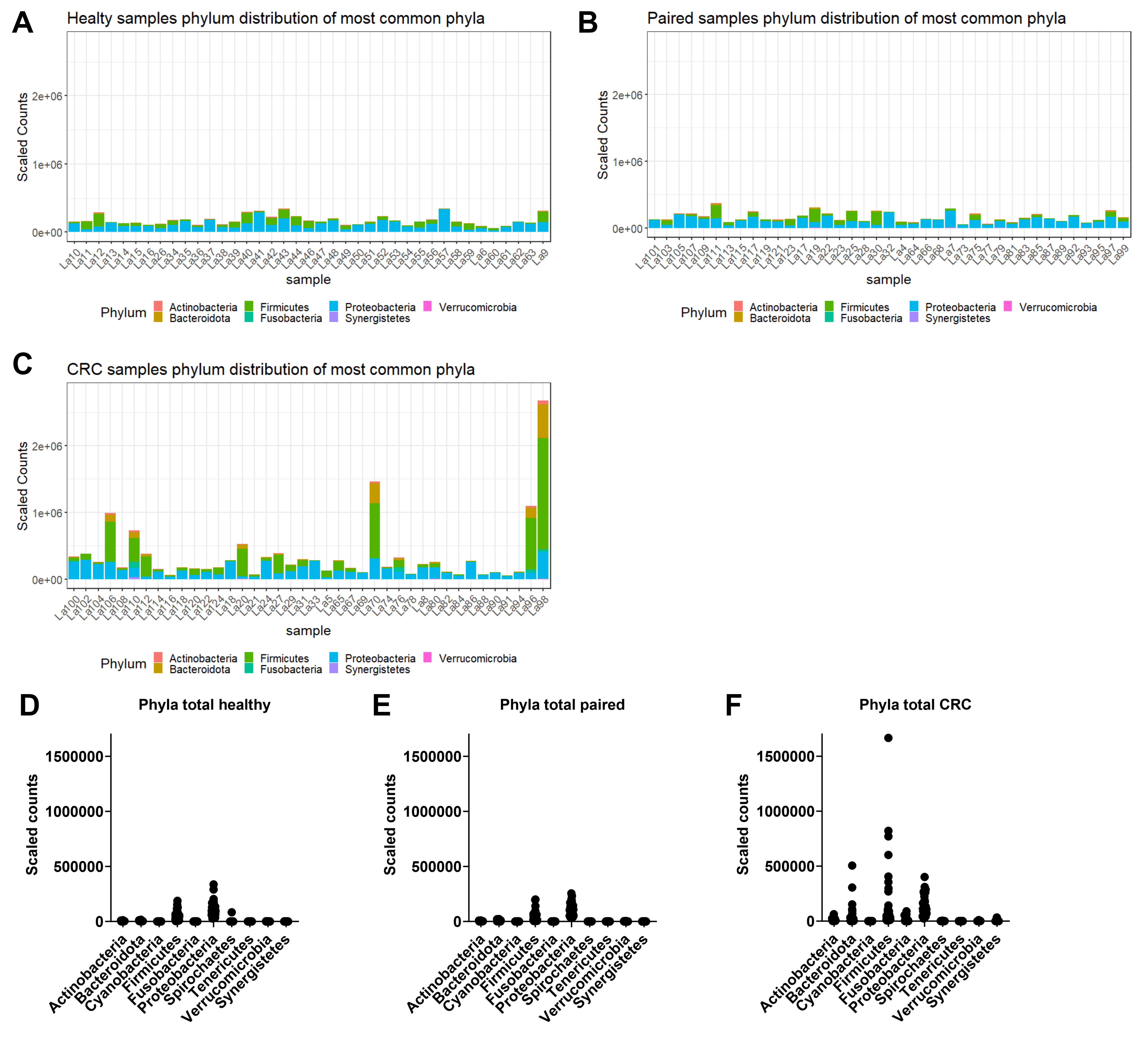


**Figure S4 –Relative abundance of bacteria in healthy, paired normal, and CRC tissue**

**A+B+C)** Scaled counts were assigned to the seven most dominant phyla for each sample in healthy (A), paired normal (B), and CRC tissue (C), respectively. **D+E+F)** Scaled counts were assigned to the ten most dominant phyla across all samples in healthy (D), paired normal (E), and CRC tissue (F), respectively.


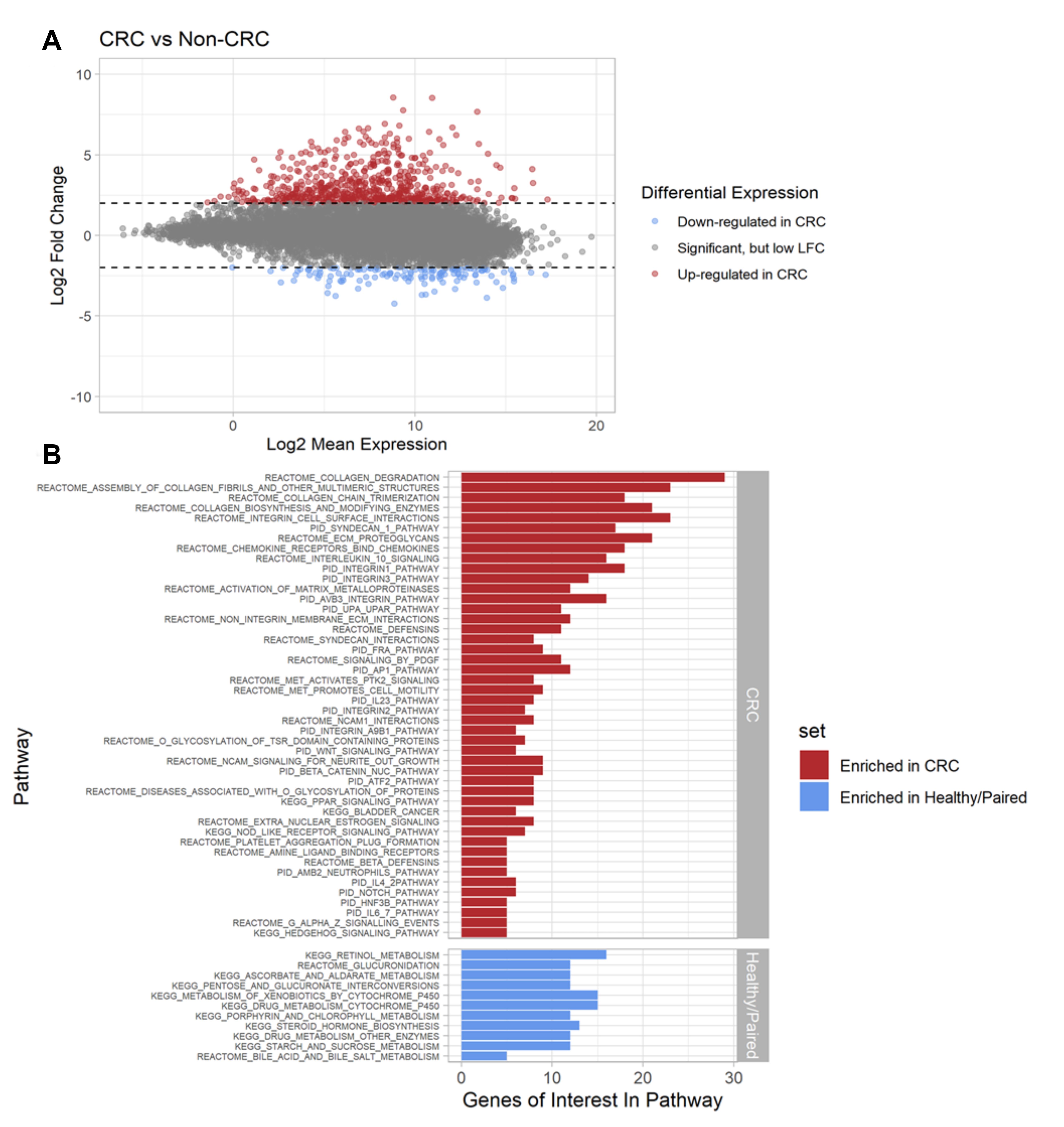


**Figure S5 - Differentially expressed genes (DEGs) and enriched biological pathways in CRC and non-CRC**

**A)** MA plot showing the distribution of significantly differentiated genes between CRC and Non-CRC (healthy and paired samples). Colouring highlights the 20 most significant DEGs with an adjusted p-value less than 0.05 and absolute log2 fold-change >2. **B)** Pathways demonstrating significant enrichment of differentially expressed genes (Fisher's exact test) for CRC or non-CRC. The Kyoto Encyclopedia of Genes and Genomes (KEGG), Pathway Interaction Database (PID), and REACTOME (a database of reactions, pathways, and biological processes) databases were used to identify pathways.


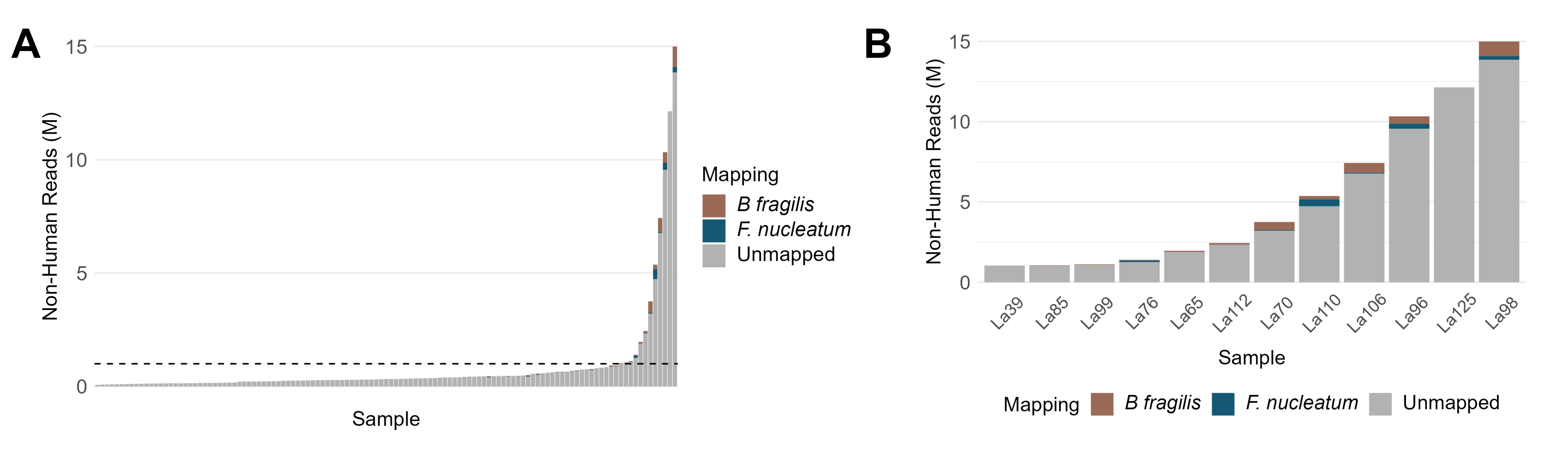


**Figure S6 – Bacterial gene expression in CRC samples**

**A)** Distribution of non-human reads in CRC samples aligned to *Bacteroides fragilis* (red), *Fusobacterium nucleatum* (blue), and other bacteria (grey). The dashed line represents a cutoff to filter out samples with reads < 1M. **B)** Distribution of non-human reads in CRC samples with more than 1M reads aligning to *B. fragilis* (red), *F. nucleatum* (blue), and other bacteria (grey).

**
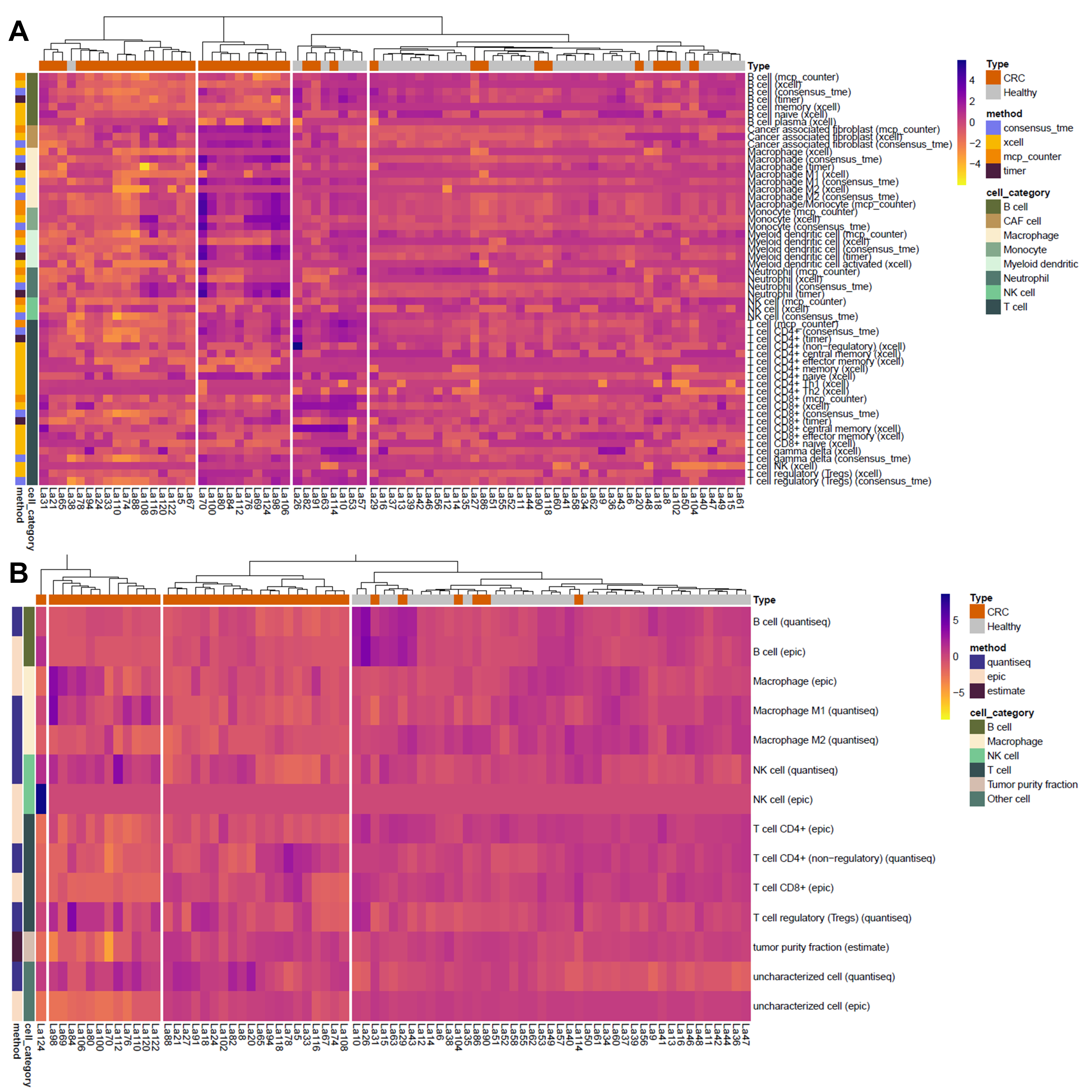
**

**Figure S7 - Clustering of samples according to immune cell infiltration**

**A+B)** Heatmaps showing immune cell profiles in CRC and healthy tissue, presented as normalized scores from the concensus_tme, xcell, mcp_counter, and timer immune scoring systems (A), and fractions from the quantisec, epic, and estimate immune scoring systems (B). Colouring from yellow (-4) to purple (4) indicates the degree of infiltration, where purple is high infiltration.
