# Supplementary material for: Biofilms and core pathogens shape the tumor microenvironment and immune phenotype in colorectal cancer
Source: Gut Microbes. 2024 May 10;16(1):2350156. doi: 10.1080/19490976.2024.2350156 (PMC11093030; doi:10.1080/19490976.2024.2350156)
Supplement: Supplemental Material [file KGMI_A_2350156_SM7231.zip › Supplemental MM 2 clean.docx]

**Supplemental Material and Methods**

**Fluorescence *in situ* hybridisation (FISH)**

PNA-FISH was carried out according to a standard in-house protocol with a few adjustments ^1^. FFPE samples were sectioned onto glass slides before standard xylene-deparaffinisation. The hybridisation buffer was prepared according to Stender *et* al. ^2^ with a final concentration of 250 nM for each probe. Samples were covered with 30 µL hybridisations buffer with a mixture of either all three probes or the specific probes (Bfrag-998 and FUS714) and left for incubation for one and a half hours at 56° C. Samples were then washed in a pre-warmed (56° C) washing buffer (AdvanDx, USA) for 30 minutes and left to dry for 15 minutes. Samples were counterstained with 0.3 µM 4′,6-diamidino-2-phenylindole (DAPI; Life Technologies, OR, USA) for 15 minutes before rinsing with cold phosphate-buffered saline pH = 7.5 (Panum Institute Substrate Department, University of Copenhagen, DK). The samples were left to dry before an antifade reagent was applied (ProLong^TM^ Gold, Thermo Fisher Scientific, UK). Finally, a cover glass (Marienfeld, DE) was added and sealed with clear nail polish.

**RNA extraction and purification**

After collection, biopsies for RNA sequencing were placed immediately in RNAlater® (Invitrogen, MA, USA) and stored for a minimum of 18-24 hours at 5 °C. Samples were subsequently centrifuged at 3000 x g for 5 minutes, the RNAlater® was removed, and the tissue was stored at -80 °C until RNA purification. Biopsies were removed from -80°C and placed immediately into 2 mL microtubes (Sarstedt, Nuembrecht, Germany) filled to ~1/3 volume with 2 and 0.1 mm diameter zirconia beads (Biospec, OK, USA) on ice. Eight hundred microliters of ice-cold Trizol (Invitrogen, MA, USA) containing 10 uL/mL β-mercaptoethanol (Sigma-Aldrich, MO, USA) was added to each tube. Samples were homogenised 3 x 30s at 7000 power in a MagnaLyzer® (Roche Diagnostics, Basel, Schweiz) and placed on ice for ~1 minute between each homogenisation. One-hundred sixty microliters of chloroform (Sigma-Aldrich, MO, USA) was added, and the tubes were shaken by hand for 45 s. Samples were spun down at 13.000 x g at 4°C for 15 min. The aqueous phase was collected in a 1.5 mL Eppendorf tube. Four hundred microliters of cold isopropanol (Sigma-Aldrich, MO, USA) and 2uL of linear acrylamide (ThermoFisher, MA, USA) were added to each sample. Tubes were then inverted 4-6 times and incubated at -20 °C for 60-90 minutes. Samples were spun down again as previously, and the supernatant was removed. The pellet was washed twice with 900uL of freshly prepared and ice-cold 80 % ethanol. After the second wash, the ethanol was removed, and the samples were air-dried for ~5-10 minutes to evaporate excess ethanol. The pellet was then resuspended in 20 uL of nuclease-free water. The concentration and purity of extracted RNA were assessed with a Nanodrop spectrophotometer (ThermoFisher, MA, USA). The purified RNA was stored at -80°C.

**Ribosomal RNA depletion**

Ribosomal RNA (rRNA) depletion was performed using the riboPOOL™ kit (siTOOls Biotech, Germany). One microgram of purified RNA was used as input, if available. If one microgram in 15uL water was not possible due to low concentration, 15 uL of the purified RNA was used as input. The protocol was performed as described in the riboPOOLKitManual_V1.3. The riboPOOL used for the depletion was a 100:1 combination of the Human riboPOOL (riboPOOL_054 ) and Pan-Prokaryote riboPOOL (riboPOOL_003). Eighty microliters of rRNA-depleted RNA were treated with RQ1 RNAse-free DNAse (Promega, USA) (10uL DNAse + 10uL buffer) per sample and incubated for 30 min at 37°C. The rRNA-depleted and DNAse-treated RNA was then cleaned with the Zymo RNA Clean and Concentrate-5 kit (Zymo Technologies, USA) and eluted in 8 uL nuclease-free water.

**Library preparation and sequencing**

One hundred nanograms of rRNA-depleted, DNAse-treated RNA in 5uL water was used as input to the NEB Ultra II directional library-preparation kit (New England BioLabs, MA, USA). If the concentration was less than this, 5uL of the rRNA-depleted, DNAse-treated RNA was used. The protocol was performed as described in the manual for rRNA-depleted RNA. Ten or twelve PCR cycles were used for the final enrichment step for samples with inputs of 100 ng or less, respectively. Quality and concentration of final libraries were measured by Qubit (1x dsDNA kit; Invitrogen, MA, USA) and Bioanalyzer (DNA High Sensitivity Chip; Agilent, CA, USA). Samples were pooled in equimolar amounts, cleaned with the 1.8x HighPrep™ PCR beads (Magbio, Lusanne, Schweiz), and sequenced on an Illumina NovaSeq 6000 instrument. Samples 1-33 and 34-118 were sequenced in S2, and S4 flow cells, respectively, with v1.5 reagents and 150 PE reads.

**Preliminary processing of raw RNA sequencing data**

Raw sequencing data (bcl. files) were demultiplexed into forward and reverse reads for each sample using bcl2fastq v2.20.0 from Illumina and concatenated across lanes. Cutadapt v3.4 ^3^ was used to trim adapters and filter out short reads (maximum error rate = 0.005, minimum length = 33, minimum overlap = 7). rRNA reads were removed with sortmeRNA v4.3.4 ^4^ using all of the included databases. The rRNA-depleted reads were then aligned to the human reference genome (GRCh38.p13, Ensembl release 106, primary assembly, build: GCA_000001405.28) with bwa-mem v0.7.17 ^5^. Reads mapping to annotated, gene-level features were counted with featureCounts (parameters: –p –O –fracOverlap 0.2 -J -t gene) from subread v2.0 ^6^ using the .gtf Ensembl annotations (GRCh38.106, Ensemble release 106). Outputted files were then concatenated by columns into a final gene-count matrix.

***B. fragilis* and *F. nucleatum* transcripts in CRC samples**

A custom reference genome was created by concatenating the reference genome .fastq files of *B. fragilis* ASM1688992v1 (<https://ftp.ncbi.nlm.nih.gov/genomes/all/GCF/016/889/925/GCF_016889925.1_ASM1688992v1/>) and *F. nucleatum* subspecies *nucleatum* GCF_003019295.1 (<https://ftp.ncbi.nlm.nih.gov/genomes/all/GCF/003/019/295/GCF_003019295.1_ASM301929v1/>). Their .gtf annotations were also concatenated in the same manner. The unmapped genes generated from the main analysis were then processed and aligned against this reference. Reads were normalised using the variance stabilising transformation from the DESeq2 R package. A cutoff was applied to filter out samples with reads < 1M. Then, the gene expression was normalised for each species, and the average across all samples that passed the filter was used. Only protein-coding genes where the product had a name in the database were included.

**References**

1. Bay L, Kragh KN, Eickhardt SR, et al. Bacterial Aggregates Establish at the Edges of Acute Epidermal Wounds. *Adv Wound Care (New Rochelle)* 2018;7(4):105-13. doi: 10.1089/wound.2017.0770 [published Online First: 2018/04/21]

2. Stender H, Mollerup TA, Lund K, et al. Direct detection and identification of *Mycobacterium tuberculosis* in smear-positive sputum samples by fluorescence in situ hybridisation (FISH) using peptide nucleic acid (PNA) probes. *The international journal of tuberculosis and lung disease : the official journal of the International Union against Tuberculosis and Lung Disease* 1999;3(9):830-7. [published Online First: 1999/09/17]

3. Martin M. Cutadapt removes adapter sequences from high-throughput sequencing reads. *2011* 2011;17(1):3. doi: 10.14806/ej.17.1.200 [published Online First: 2011-08-02]

4. Kopylova E, Noé L, Touzet H. SortMeRNA: fast and accurate filtering of ribosomal RNAs in metatranscriptomic data. *Bioinformatics* 2012;28(24):3211-7. doi: 10.1093/bioinformatics/bts611 [published Online First: 2012/10/17]

5. Li H. Aligning sequence reads, clone sequences and assembly contigs with BWA-MEM. *arXiv: Genomics* 2013 doi: <https://doi.org/10.48550/arXiv.1303.3997>

6. Liao Y, Smyth GK, Shi W. The Subread aligner: fast, accurate and scalable read mapping by seed-and-vote. *Nucleic Acids Res* 2013;41(10):e108. doi: 10.1093/nar/gkt214 [published Online First: 2013/04/06]
