# Supplementary material for: Biofilms and core pathogens shape the tumor microenvironment and immune phenotype in colorectal cancer
Source: Gut Microbes. 2024 May 10;16(1):2350156. doi: 10.1080/19490976.2024.2350156 (PMC11093030; doi:10.1080/19490976.2024.2350156)
Supplement: Supplemental Material [file KGMI_A_2350156_SM7231.zip › Supplemental Tables 2.docx]

**Supplemental Tables**

| Characteristics | CRC, N = 37 | Healthy, N = 40 | P-value |
| --- | --- | --- | --- |
| Sex, male | 22 (61.1) | 25 (62.5) | >0.99 |
| Age, median (range)  Weight in Kg., median (range)  BMI, median (range)  Missing information (BMI) | 76 (47 – 90)  74 (46 – 119)  24.8 (16.7 – 41.2)  2 | 68.5 (46 –86)  76 (49 – 121)  25.8 (17.6 – 42.9)  2 | 0.05  0.71  0.40 |
| Anatomic location of sampling (%)  Left-sided  Right-sided  Missing information | 28 (75.7)  9 (24.3) | 22 (56.4)  17 (43.6)  1 | 0.09 |
| T stage (%)  1  2  3  4  Missing information | 4 (10.8)  7 (18.9)  13 (35.1)  5 (13.5)  8 | N/A  N/A  N/A  N/A | N/A  N/A  N/A  N/A |
| Lymph node metastases, N (%)  Distant metastases, M (%)  Missing information, N/M (%)  pMMR/dMMR | 12 (32.4)  7 (18.9)  3/2  35/2 | N/A  N/A  N/A | N/A  N/A  N/A |
| ASA score (%)  1  2  3  Missing information | 2 (5.4)  24 (64.9)  11 (29.7) | 6 (15.0)  20 (50.0)  1 (2.5)  13 | <0.01 |
| Smoking (%)  Yes  No  Previously  Missing information | 9 (24.3)  12 (32.4)  15 (40.5)  1 | 3 (7.5)  14 (35.0)  15 (37.0)  8 | 0.46^*^ |
| Diabetes (%)  DM1  DM2  Missing information | 1 (2.7)  9 (24.3) | 0 (0)  6 (15.0)  6 | 0.40* |

**Table S1 - Characteristics of included patients with CRC and healthy persons.**

ASA, American Society of Anesthesiologists. BMI, Body Mass Index (Kg/m^2). DM, Diabetes mellitus. N/A, not applicable. ^*^Current and previous smoking and DM1 and DM2 have been pooled for statistical analysis. Continuous data was tested with a two-sided student t-test or Mann-Whitney Test, and categorical data was tested with a chi-square test. A p-value ≤ 0.05 was considered statistically significant.

| **Species** | **Gene** | **Annotation** | **Name** | **Product** |
| --- | --- | --- | --- | --- |
| *B_fragilis* | I6J55_RS00210 | NZ_CP069563.1 | rnpB | RNase P RNA component class A |
| *B_fragilis* | I6J55_RS15105 | NZ_CP069563.1 | tuf | elongation factor Tu |
| *B_fragilis* | I6J55_RS15190 | NZ_CP069563.1 | fusA | elongation factor G |
| *B_fragilis* | I6J55_RS08765 | NZ_CP069563.1 | ssrA | transfer-messenger RNA |
| *B_fragilis* | I6J55_RS14165 | NZ_CP069563.1 | pckA | phosphoenolpyruvate carboxykinase (ATP) |
| *B_fragilis* | I6J55_RS15145 | NZ_CP069563.1 | rpoB | DNA-directed RNA polymerase subunit beta |
| *B_fragilis* | I6J55_RS15150 | NZ_CP069563.1 | rpoC | DNA-directed RNA polymerase subunit beta' |
| *B_fragilis* | I6J55_RS15120 | NZ_CP069563.1 | nusG | transcription termination/antitermination protein NusG |
| *B_fragilis* | I6J55_RS09490 | NZ_CP069563.1 | gap | type I glyceraldehyde-3-phosphate dehydrogenase |
| *B_fragilis* | I6J55_RS19030 | NZ_CP069563.1 | groL | chaperonin GroEL |
| *B_fragilis* | I6J55_RS19300 | NZ_CP069563.1 | nifJ | pyruvate:ferredoxin (flavodoxin) oxidoreductase |
| *B_fragilis* | I6J55_RS15300 | NZ_CP069563.1 | secY | preprotein translocase subunit SecY |
| *B_fragilis* | I6J55_RS08415 | NZ_CP069563.1 | dnaK | molecular chaperone DnaK |
| *B_fragilis* | I6J55_RS15075 | NZ_CP069563.1 | xerC | tyrosine recombinase XerC |
| *B_fragilis* | I6J55_RS12720 | NZ_CP069563.1 | fabF | beta-ketoacyl-ACP synthase II |
| *B_fragilis* | I6J55_RS12520 | NZ_CP069563.1 | infB | translation initiation factor IF-2 |
| *B_fragilis* | I6J55_RS16120 | NZ_CP069563.1 | tsf | translation elongation factor Ts |
| *B_fragilis* | I6J55_RS01225 | NZ_CP069563.1 | ppdK | pyruvate, phosphate dikinase |
| *B_fragilis* | I6J55_RS14715 | NZ_CP069563.1 | pnp | polyribonucleotide nucleotidyltransferase |
| *B_fragilis* | I6J55_RS15305 | NZ_CP069563.1 | map | type I methionyl aminopeptidase |
| *F_nucleatum* | C7Y58_RS00300 | NZ_CP028101.1 | tuf | elongation factor Tu |
| *F_nucleatum* | C7Y58_RS09590 | NZ_CP028101.1 | rnpB | RNase P RNA component class A |
| *F_nucleatum* | C7Y58_RS00305 | NZ_CP028101.1 | fusA | elongation factor G |
| *F_nucleatum* | C7Y58_RS08880 | NZ_CP028101.1 | nifJ | pyruvate:ferredoxin (flavodoxin) oxidoreductase |
| *F_nucleatum* | C7Y58_RS06320 | NZ_CP028101.1 | gap | type I glyceraldehyde-3-phosphate dehydrogenase |
| *F_nucleatum* | C7Y58_RS02690 | NZ_CP028101.1 | rpoC | DNA-directed RNA polymerase subunit beta' |
| *F_nucleatum* | C7Y58_RS04375 | NZ_CP028101.1 | pflB | formate C-acetyltransferase |
| *F_nucleatum* | C7Y58_RS01655 | NZ_CP028101.1 | dnaK | molecular chaperone DnaK |
| *F_nucleatum* | C7Y58_RS02685 | NZ_CP028101.1 | rpoB | DNA-directed RNA polymerase subunit beta |
| *F_nucleatum* | C7Y58_RS02970 | NZ_CP028101.1 | ftsH | ATP-dependent zinc metalloprotease FtsH |
| *F_nucleatum* | C7Y58_RS02945 | NZ_CP028101.1 | ahpC | alkyl hydroperoxide reductase subunit C |
| *F_nucleatum* | C7Y58_RS06435 | NZ_CP028101.1 | groL | chaperonin GroEL |
| *F_nucleatum* | C7Y58_RS03290 | NZ_CP028101.1 | rny | ribonuclease Y |
| *F_nucleatum* | C7Y58_RS03155 | NZ_CP028101.1 | clpB | ATP-dependent chaperone ClpB |
| *F_nucleatum* | C7Y58_RS01315 | NZ_CP028101.1 | eno | phosphopyruvate hydratase |
| *F_nucleatum* | C7Y58_RS05850 | NZ_CP028101.1 | ssrA | transfer-messenger RNA |
| *F_nucleatum* | C7Y58_RS01045 | NZ_CP028101.1 | pnp | polyribonucleotide nucleotidyltransferase |
| *F_nucleatum* | C7Y58_RS01095 | NZ_CP028101.1 | secA | preprotein translocase subunit SecA |
| *F_nucleatum* | C7Y58_RS02765 | NZ_CP028101.1 | infB | translation initiation factor IF-2 |
| *F_nucleatum* | C7Y58_RS04850 | NZ_CP028101.1 | atpD | F0F1 ATP synthase subunit beta |

**Table S2 – Top 20 species-specific expressed genes in CRC samples.**

| **Specific counts** | **Immune clusters (1-4)** | **p-value**  **(including healthy and paired normal samples)** | **p-value**  **(including healthy samples)** |
| --- | --- | --- | --- |
| Bacteria | Cluster 1 vs Cluster 2 | ns | ns |
| Bacteria | Cluster 1 vs Cluster 3 | ns | ns |
| Bacteria | Cluster 1 vs Cluster 4 | ns | ns |
| Bacteria | Cluster 2 vs Cluster 3 | 0.007 | ns |
| Bacteria | Cluster 2 vs Cluster 4 | ns | ns |
| Bacteria | Cluster 3 vs Cluster 4 | ns | ns |
| *F. nucleatum* | Cluster 1 vs Cluster 2 | ns | ns |
| *F. nucleatum* | Cluster 1 vs Cluster 3 | 0.005 | 0.034 |
| *F. nucleatum* | Cluster 1 vs Cluster 4 | <0.001 | ns |
| *F. nucleatum* | Cluster 2 vs Cluster 3 | 0.012 | 0.037 |
| *F. nucleatum* | Cluster 2 vs Cluster 4 | 0.009 | ns |
| *F. nucleatum* | Cluster 3 vs Cluster 4 | ns | ns |
| *B. fragilis* | Cluster 1 vs Cluster 2 | ns | ns |
| *B. fragilis* | Cluster 1 vs Cluster 3 | 0.005 | 0.037 |
| *B. fragilis* | Cluster 1 vs Cluster 4 | ns | ns |
| *B. fragilis* | Cluster 2 vs Cluster 3 | ns | 0.009 |
| *B. fragilis* | Cluster 2 vs Cluster 4 | ns | ns |
| *B. fragilis* | Cluster 3 vs Cluster 4 | ns | ns |

**Table S3 – Difference in bacterial and species-specific activity between immune clusters 1-4.**

Four immune clusters were identified, separating healthy samples (Clusters 1 and 2) and CRC samples (Clusters 3 and 4) with high (Clusters 2 and 3) and low or normal immune scores (Clusters 1 and 4). A comparison of bacterial or species-specif activity across clusters was tested using a non-parametric Kruskal-Wallace test followed by a post-hoc Dunn test. A p-value < 0.05 was concidered significant. ns = not significant.

| Cell_type | Estimate frac | Estimate score | timer | consensus_tme | xcell | mcp_counter | epic | quantiseq |
| --- | --- | --- | --- | --- | --- | --- | --- | --- |
| B cell | NA | NA | ns | ns | ns | ns | ns | NA |
| B cell memory | NA | NA | NA | NA | ns | NA | NA | NA |
| B cell naive | NA | NA | NA | NA | ns | NA | NA | NA |
| B cell plasma | NA | NA | NA | NA | ns | NA | NA | NA |
| Cancer associated fibroblast | NA | NA | NA | ns | ns | ns | ns | NA |
| Class-switched memory B cell | NA | NA | NA | NA | ns | NA | NA | NA |
| Common lymphoid progenitor | NA | NA | NA | NA | ns | NA | NA | NA |
| Common myeloid progenitor | NA | NA | NA | NA | ns | NA | NA | NA |
| cytotoxicity score | NA | NA | NA | ns | NA | ns | NA | NA |
| Endothelial cell | NA | NA | NA | ** | **** | *** | * | NA |
| Eosinophil | NA | NA | NA | ns | ns | NA | NA | NA |
| Granulocyte-monocyte progenitor | NA | NA | NA | NA | ns | NA | NA | NA |
| Hematopoietic stem cell | NA | NA | NA | NA | *** | NA | NA | NA |
| immune score | NA | ns | NA | ns | ns | NA | NA | NA |
| Macrophage | NA | NA | ns | ns | ns | NA | ns | NA |
| Macrophage M1 | NA | NA | NA | ns | ns | NA | NA | NA |
| Macrophage M2 | NA | NA | NA | ns | ns | NA | NA | NA |
| **Macrophage/Monocyte** | **NA** | **NA** | **NA** | **NA** | **NA** | ****** | **NA** | **NA** |
| Mast cell | NA | NA | NA | NA | ns | NA | NA | NA |
| MAST cell | NA | NA | NA | ns | NA | NA | NA | NA |
| microenvironment score | NA | NA | NA | NA | ns | NA | NA | NA |
| Monocyte | NA | NA | NA | ns | * | ** | NA | NA |
| Myeloid dendritic cell | NA | NA | ** | ns | ns | ns | NA | NA |
| **Myeloid dendritic cell activated** | **NA** | **NA** | **NA** | **NA** | ***** | **NA** | **NA** | **NA** |
| Neutrophil | NA | NA | ** | ns | * | ns | NA | NA |
| NK cell | NA | NA | NA | ns | ns | ns | ns | NA |
| Plasmacytoid dendritic cell | NA | NA | NA | NA | ns | NA | NA | NA |
| stroma score | NA | ** | NA | NA | * | NA | NA | NA |
| T cell | NA | NA | NA | NA | NA | ns | NA | NA |
| T cell CD4+ | NA | NA | ns | ns | NA | NA | ns | NA |
| T cell CD4+ (non-regulatory) | NA | NA | NA | NA | ns | NA | NA | NA |
| T cell CD4+ central memory | NA | NA | NA | NA | ns | NA | NA | NA |
| T cell CD4+ effector memory | NA | NA | NA | NA | ns | NA | NA | NA |
| T cell CD4+ memory | NA | NA | NA | NA | ns | NA | NA | NA |
| T cell CD4+ naive | NA | NA | NA | NA | ns | NA | NA | NA |
| T cell CD4+ Th1 | NA | NA | NA | NA | ns | NA | NA | NA |
| T cell CD4+ Th2 | NA | NA | NA | NA | ns | NA | NA | NA |
| T cell CD8+ | NA | NA | ns | ns | ns | ns | * | NA |
| T cell CD8+ central memory | NA | NA | NA | NA | ns | NA | NA | NA |
| T cell CD8+ effector memory | NA | NA | NA | NA | ns | NA | NA | NA |
| T cell CD8+ naive | NA | NA | NA | NA | ns | NA | NA | NA |
| T cell gamma delta | NA | NA | NA | ns | ns | NA | NA | NA |
| T cell NK | NA | NA | NA | NA | ns | NA | NA | NA |
| **T cell regulatory (Tregs)** | **NA** | **NA** | **NA** | **ns** | ******* | **NA** | **NA** | **NA** |
| **tumor purity fraction** | ******* | **NA** | **NA** | **NA** | **NA** | **NA** | **NA** | **NA** |
| **tumor purity score** | **NA** | ***** | **NA** | **NA** | **NA** | **NA** | **NA** | **NA** |
| uncharacterized cell | NA | NA | NA | NA | NA | NA | ns | NA |

**Table S4 – Bacterial counts impacting specific immune cells across the seven methods that score immune cell infiltration.**

NA = Not applicable because the method does not report that type of immune cell. ns = not significant. * = p<0.05, ** = p<0.01, *** p<0.001, **** p<0.0001. Statistical comparison was carried out with Ordinary Least Squares regression to determine which independent variables (sample type, bacterial count, read count) explain the dependent outcome variable (the immune score). Highlighted rows with bold text indicate immune cell sub-populations only affected in CRC tissue by bacterial counts. A p-value ≤ 0.05 was considered statistically significant.

| Cell_type | Estimate_frac | Estimate_score | Timer | consensus_tme | xcell | mcp_counter | epic | quantiseq |
| --- | --- | --- | --- | --- | --- | --- | --- | --- |
| B cell | NA | NA | ns | ns | ns | ns | ns | NA |
| B cell memory | NA | NA | NA | NA | ns | NA | NA | NA |
| B cell naive | NA | NA | NA | NA | ns | NA | NA | NA |
| B cell plasma | NA | NA | NA | NA | ns | NA | NA | NA |
| Cancer associated fibroblast | NA | NA | NA | ns | ns | ns | ns | NA |
| Class-switched memory B cell | NA | NA | NA | NA | ns | NA | NA | NA |
| Common lymphoid progenitor | NA | NA | NA | NA | ns | NA | NA | NA |
| Common myeloid progenitor | NA | NA | NA | NA | ns | NA | NA | NA |
| cytotoxicity score | NA | NA | NA | ns | NA | ns | NA | NA |
| Endothelial cell | NA | NA | NA | ns | ns | ns | ns | NA |
| Eosinophil | NA | NA | NA | ns | ns | NA | NA | NA |
| Granulocyte-monocyte progenitor | NA | NA | NA | NA | ns | NA | NA | NA |
| Hematopoietic stem cell | NA | NA | NA | NA | ns | NA | NA | NA |
| immune score | NA | ns | NA | ns | ns | NA | NA | NA |
| Macrophage | NA | NA | ns | ns | ns | NA | ns | NA |
| Macrophage M1 | NA | NA | NA | ns | ns | NA | NA | NA |
| Macrophage M2 | NA | NA | NA | ns | ns | NA | NA | NA |
| Macrophage/Monocyte | NA | NA | NA | NA | NA | ns | NA | NA |
| Mast cell | NA | NA | NA | NA | ns | NA | NA | NA |
| MAST cell | NA | NA | NA | ns | NA | NA | NA | NA |
| microenvironment score | NA | NA | NA | NA | ns | NA | NA | NA |
| Monocyte | NA | NA | NA | ns | ns | ns | NA | NA |
| Myeloid dendritic cell | NA | NA | ns | ns | ns | ns | NA | NA |
| Myeloid dendritic cell activated | NA | NA | NA | NA | ns | NA | NA | NA |
| **Neutrophil** | **NA** | **NA** | **ns** | **ns** | ***** | **ns** | **NA** | **NA** |
| NK cell | NA | NA | NA | ns | ns | ns | ns | NA |
| Plasmacytoid dendritic cell | NA | NA | NA | NA | ns | NA | NA | NA |
| stroma score | NA | ns | NA | NA | ns | NA | NA | NA |
| T cell | NA | NA | NA | NA | NA | ns | NA | NA |
| T cell CD4+ | NA | NA | ns | ns | NA | NA | ns | NA |
| T cell CD4+ (non-regulatory) | NA | NA | NA | NA | ns | NA | NA | NA |
| T cell CD4+ central memory | NA | NA | NA | NA | ns | NA | NA | NA |
| T cell CD4+ effector memory | NA | NA | NA | NA | ns | NA | NA | NA |
| T cell CD4+ memory | NA | NA | NA | NA | ns | NA | NA | NA |
| T cell CD4+ naive | NA | NA | NA | NA | ns | NA | NA | NA |
| T cell CD4+ Th1 | NA | NA | NA | NA | ns | NA | NA | NA |
| T cell CD4+ Th2 | NA | NA | NA | NA | ns | NA | NA | NA |
| T cell CD8+ | NA | NA | ns | ns | ns | ns | ns | NA |
| T cell CD8+ central memory | NA | NA | NA | NA | ns | NA | NA | NA |
| T cell CD8+ effector memory | NA | NA | NA | NA | ns | NA | NA | NA |
| T cell CD8+ naive | NA | NA | NA | NA | ns | NA | NA | NA |
| T cell gamma delta | NA | NA | NA | ns | ns | NA | NA | NA |
| T cell NK | NA | NA | NA | NA | ns | NA | NA | NA |
| T cell regulatory (Tregs) | NA | NA | NA | ns | ns | NA | NA | NA |
| **tumor purity fraction** | ***** | **NA** | **NA** | **NA** | **NA** | **NA** | **NA** | **NA** |
| tumor purity score | NA | ns | NA | NA | NA | NA | NA | NA |
| uncharacterized cell | NA | NA | NA | NA | NA | NA | ns | NA |

**Table S5 – *Bacteroides fragilis* impacting specific immune cells across the seven methods that score immune cell infiltration.**

NA = Not applicable because the method does not report that type of immune cell. ns = not significant. * = p<0.05. Statistical comparison was carried out with Ordinary Least Squares regression to determine which independent variables (sample type, bacterial count, read count) explain the dependent outcome variable (the immune score). Highlighted rows with bold text indicate immune cell sub-populations only affected in CRC tissue by *B. fragilis*. A p-value ≤ 0.05 was considered statistically significant.

| Cell_type | Estimate_frac | Estimate_score | Timer | consensus_tme | xcell | mcp_counter | epic | quantiseq |
| --- | --- | --- | --- | --- | --- | --- | --- | --- |
| B cell | NA | NA | ns | ns | ns | ns | ns | NA |
| B cell memory | NA | NA | NA | NA | ns | NA | NA | NA |
| B cell naive | NA | NA | NA | NA | ns | NA | NA | NA |
| B cell plasma | NA | NA | NA | NA | ns | NA | NA | NA |
| Cancer associated fibroblast | NA | NA | NA | ns | ns | ns | ns | NA |
| Class-switched memory B cell | NA | NA | NA | NA | ns | NA | NA | NA |
| Common lymphoid progenitor | NA | NA | NA | NA | ns | NA | NA | NA |
| Common myeloid progenitor | NA | NA | NA | NA | ns | NA | NA | NA |
| cytotoxicity score | NA | NA | NA | ns | NA | ns | NA | NA |
| Endothelial cell | NA | NA | NA | ns | ns | ns | ns | NA |
| Eosinophil | NA | NA | NA | ns | ns | NA | NA | NA |
| Granulocyte-monocyte progenitor | NA | NA | NA | NA | ns | NA | NA | NA |
| Hematopoietic stem cell | NA | NA | NA | NA | ns | NA | NA | NA |
| immune score | NA | ns | NA | ns | ns | NA | NA | NA |
| Macrophage | NA | NA | ns | ns | ns | NA | ns | NA |
| Macrophage M1 | NA | NA | NA | ns | ns | NA | NA | NA |
| Macrophage M2 | NA | NA | NA | ns | ns | NA | NA | NA |
| Macrophage/Monocyte | NA | NA | NA | NA | NA | ns | NA | NA |
| Mast cell | NA | NA | NA | NA | ns | NA | NA | NA |
| MAST cell | NA | NA | NA | ns | NA | NA | NA | NA |
| microenvironment score | NA | NA | NA | NA | ns | NA | NA | NA |
| Monocyte | NA | NA | NA | ns | ns | ns | NA | NA |
| Myeloid dendritic cell | NA | NA | ns | ns | ns | ns | NA | NA |
| Myeloid dendritic cell activated | NA | NA | NA | NA | ns | NA | NA | NA |
| Neutrophil | NA | NA | ns | ns | ns | ns | NA | NA |
| NK cell | NA | NA | NA | ns | ns | ns | ns | NA |
| Plasmacytoid dendritic cell | NA | NA | NA | NA | ns | NA | NA | NA |
| stroma score | NA | ns | NA | NA | ns | NA | NA | NA |
| T cell | NA | NA | NA | NA | NA | ns | NA | NA |
| T cell CD4+ | NA | NA | ns | ns | NA | NA | ns | NA |
| T cell CD4+ (non-regulatory) | NA | NA | NA | NA | ns | NA | NA | NA |
| T cell CD4+ central memory | NA | NA | NA | NA | ns | NA | NA | NA |
| T cell CD4+ effector memory | NA | NA | NA | NA | ns | NA | NA | NA |
| T cell CD4+ memory | NA | NA | NA | NA | ns | NA | NA | NA |
| T cell CD4+ naive | NA | NA | NA | NA | ns | NA | NA | NA |
| T cell CD4+ Th1 | NA | NA | NA | NA | ns | NA | NA | NA |
| T cell CD4+ Th2 | NA | NA | NA | NA | ns | NA | NA | NA |
| T cell CD8+ | NA | NA | ns | ns | ns | ns | ns | NA |
| T cell CD8+ central memory | NA | NA | NA | NA | ns | NA | NA | NA |
| T cell CD8+ effector memory | NA | NA | NA | NA | ns | NA | NA | NA |
| T cell CD8+ naive | NA | NA | NA | NA | ns | NA | NA | NA |
| T cell gamma delta | NA | NA | NA | ns | ns | NA | NA | NA |
| T cell NK | NA | NA | NA | NA | ns | NA | NA | NA |
| T cell regulatory (Tregs) | NA | NA | NA | ns | ns | NA | NA | NA |
| tumor purity fraction | ns | NA | NA | NA | NA | NA | NA | NA |
| tumor purity score | NA | ns | NA | NA | NA | NA | NA | NA |
| uncharacterized cell | NA | NA | NA | NA | NA | NA | ns | NA |

**Table S6 – *Fusobacterium nucleatum* impacting specific immune cells across the seven methods that score immune cell infiltration.**

NA = Not applicable because the method does not report that type of immune cell. ns = not significant. Statistical comparison was carried out with Ordinary Least Squares regression to determine which independent variables (sample type, bacterial count, read count) explain the dependent outcome variable (the immune score). A p-value ≤ 0.05 was considered statistically significant.

| Cell_type | Estimate  _frac | Estimate  _score | Timer | Consensus  _tme | xcell | Mcp  _counter | epic | quantiseq |
| --- | --- | --- | --- | --- | --- | --- | --- | --- |
| B cell | NA | NA | ns | ns | ns | ns | ns | NA |
| B cell memory | NA | NA | NA | NA | ns | NA | NA | NA |
| B cell naive | NA | NA | NA | NA | ns | NA | NA | NA |
| B cell plasma | NA | NA | NA | NA | ns | NA | NA | NA |
| Cancer associated fibroblast | NA | NA | NA | ns | ns | ns | ns | NA |
| Class-switched memory B cell | NA | NA | NA | NA | ns | NA | NA | NA |
| Common lymphoid progenitor | NA | NA | NA | NA | ns | NA | NA | NA |
| Common myeloid progenitor | NA | NA | NA | NA | ns | NA | NA | NA |
| cytotoxicity score | NA | NA | NA | ns | NA | ns | NA | NA |
| Endothelial cell | NA | NA | NA | ** | *** | ** | * | NA |
| **Eosinophil** | **NA** | **NA** | **NA** | ***** | **ns** | **NA** | **NA** | **NA** |
| Granulocyte-monocyte progenitor | NA | NA | NA | NA | ns | NA | NA | NA |
| **Hematopoietic stem cell** | **NA** | **NA** | **NA** | **NA** | ****** | **NA** | **NA** | **NA** |
| **immune score** | **NA** | ****** | **NA** | ***** | **ns** | **NA** | **NA** | **NA** |
| Macrophage | NA | NA | ns | * | ns | NA | ns | NA |
| Macrophage M1 | NA | NA | NA | ns | ns | NA | NA | NA |
| **Macrophage M2** | **NA** | **NA** | **NA** | ***** | ***** | **NA** | **NA** | **NA** |
| **Macrophage/Monocyte** | **NA** | **NA** | **NA** | **NA** | **NA** | ****** | **NA** | **NA** |
| Mast cell | NA | NA | NA | NA | ns | NA | NA | NA |
| MAST cell | NA | NA | NA | ns | NA | NA | NA | NA |
| **microenvironment score** | **NA** | **NA** | **NA** | **NA** | ***** | **NA** | **NA** | **NA** |
| **Monocyte** | **NA** | **NA** | **NA** | ***** | **ns** | ****** | **NA** | **NA** |
| Myeloid dendritic cell | NA | NA | *** | * | ns | ns | NA | NA |
| **Myeloid dendritic cell activated** | **NA** | **NA** | **NA** | **NA** | ******* | **NA** | **NA** | **NA** |
| Neutrophil | NA | NA | ** | ns | ns | ns | NA | NA |
| NK cell | NA | NA | NA | ns | ns | ns | ns | NA |
| Plasmacytoid dendritic cell | NA | NA | NA | NA | ns | NA | NA | NA |
| stroma score | NA | ** | NA | NA | * | NA | NA | NA |
| T cell | NA | NA | NA | NA | NA | ns | NA | NA |
| T cell CD4+ | NA | NA | ns | ns | NA | NA | ns | NA |
| T cell CD4+ (non-regulatory) | NA | NA | NA | NA | ns | NA | NA | NA |
| T cell CD4+ central memory | NA | NA | NA | NA | ns | NA | NA | NA |
| T cell CD4+ effector memory | NA | NA | NA | NA | ns | NA | NA | NA |
| T cell CD4+ memory | NA | NA | NA | NA | ns | NA | NA | NA |
| T cell CD4+ naive | NA | NA | NA | NA | ns | NA | NA | NA |
| T cell CD4+ Th1 | NA | NA | NA | NA | ns | NA | NA | NA |
| T cell CD4+ Th2 | NA | NA | NA | NA | ns | NA | NA | NA |
| T cell CD8+ | NA | NA | ns | ns | ns | ns | * | NA |
| T cell CD8+ central memory | NA | NA | NA | NA | ns | NA | NA | NA |
| T cell CD8+ effector memory | NA | NA | NA | NA | ns | NA | NA | NA |
| T cell CD8+ naive | NA | NA | NA | NA | ns | NA | NA | NA |
| T cell gamma delta | NA | NA | NA | ns | ns | NA | NA | NA |
| T cell NK | NA | NA | NA | NA | ns | NA | NA | NA |
| **T cell regulatory (Tregs)** | **NA** | **NA** | **NA** | **ns** | ******* | **NA** | **NA** | **NA** |
| **tumor purity fraction** | ******** | **NA** | **NA** | **NA** | **NA** | **NA** | **NA** | **NA** |
| **tumor purity score** | **NA** | ****** | **NA** | **NA** | **NA** | **NA** | **NA** | **NA** |
| uncharacterized cell | NA | NA | NA | NA | NA | NA | ns | NA |

**Table S7 – Bacterial counts impacting specific immune cells across the seven methods that score immune cell infiltration, excluding paired normal samples from the control group.**

NA = Not applicable because the method does not report that type of immune cell. ns = not significant. * = p<0.05, ** = p<0.01, *** p<0.001, **** p<0.0001. Statistical comparison was carried out with Ordinary Least Squares regression to determine which independent variables (sample type, bacterial count, read count) explain the dependent outcome variable (the immune score). Highlighted rows with bold text indicate immune cell sub-populations only affected in CRC tissue by bacterial counts. A p-value ≤ 0.05 was considered statistically significant.

| Cell_type | Estimate_frac | Estimate_score | Timer | consensus_tme | xcell | mcp_counter | epic | quantiseq |
| --- | --- | --- | --- | --- | --- | --- | --- | --- |
| B cell | NA | NA | ns | ns | ns | ns | ns | NA |
| B cell memory | NA | NA | NA | NA | ns | NA | NA | NA |
| B cell naive | NA | NA | NA | NA | ns | NA | NA | NA |
| B cell plasma | NA | NA | NA | NA | ns | NA | NA | NA |
| Cancer associated fibroblast | NA | NA | NA | ns | ns | ns | ns | NA |
| Class-switched memory B cell | NA | NA | NA | NA | ns | NA | NA | NA |
| Common lymphoid progenitor | NA | NA | NA | NA | ns | NA | NA | NA |
| Common myeloid progenitor | NA | NA | NA | NA | ns | NA | NA | NA |
| cytotoxicity score | NA | NA | NA | ns | NA | ns | NA | NA |
| Endothelial cell | NA | NA | NA | ns | ns | ns | ns | NA |
| Eosinophil | NA | NA | NA | ns | ns | NA | NA | NA |
| Granulocyte-monocyte progenitor | NA | NA | NA | NA | ns | NA | NA | NA |
| Hematopoietic stem cell | NA | NA | NA | NA | ns | NA | NA | NA |
| immune score | NA | ns | NA | ns | ns | NA | NA | NA |
| Macrophage | NA | NA | ns | ns | ns | NA | ns | NA |
| Macrophage M1 | NA | NA | NA | ns | ns | NA | NA | NA |
| Macrophage M2 | NA | NA | NA | ns | ns | NA | NA | NA |
| Macrophage/Monocyte | NA | NA | NA | NA | NA | ns | NA | NA |
| Mast cell | NA | NA | NA | NA | ns | NA | NA | NA |
| MAST cell | NA | NA | NA | ns | NA | NA | NA | NA |
| microenvironment score | NA | NA | NA | NA | ns | NA | NA | NA |
| Monocyte | NA | NA | NA | ns | ns | ns | NA | NA |
| Myeloid dendritic cell | NA | NA | ns | ns | ns | ns | NA | NA |
| Myeloid dendritic cell activated | NA | NA | NA | NA | ns | NA | NA | NA |
| Neutrophil | NA | NA | ns | ns | ns | ns | NA | NA |
| NK cell | NA | NA | NA | ns | ns | ns | ns | NA |
| Plasmacytoid dendritic cell | NA | NA | NA | NA | ns | NA | NA | NA |
| stroma score | NA | ns | NA | NA | ns | NA | NA | NA |
| T cell | NA | NA | NA | NA | NA | ns | NA | NA |
| T cell CD4+ | NA | NA | ns | ns | NA | NA | ns | NA |
| T cell CD4+ (non-regulatory) | NA | NA | NA | NA | ns | NA | NA | NA |
| T cell CD4+ central memory | NA | NA | NA | NA | ns | NA | NA | NA |
| T cell CD4+ effector memory | NA | NA | NA | NA | ns | NA | NA | NA |
| T cell CD4+ memory | NA | NA | NA | NA | ns | NA | NA | NA |
| T cell CD4+ naive | NA | NA | NA | NA | ns | NA | NA | NA |
| T cell CD4+ Th1 | NA | NA | NA | NA | ns | NA | NA | NA |
| T cell CD4+ Th2 | NA | NA | NA | NA | ns | NA | NA | NA |
| T cell CD8+ | NA | NA | ns | ns | ns | ns | ns | NA |
| T cell CD8+ central memory | NA | NA | NA | NA | ns | NA | NA | NA |
| T cell CD8+ effector memory | NA | NA | NA | NA | ns | NA | NA | NA |
| T cell CD8+ naive | NA | NA | NA | NA | ns | NA | NA | NA |
| T cell gamma delta | NA | NA | NA | ns | ns | NA | NA | NA |
| T cell NK | NA | NA | NA | NA | ns | NA | NA | NA |
| T cell regulatory (Tregs) | NA | NA | NA | ns | ns | NA | NA | NA |
| tumor purity fraction | ns | NA | NA | NA | NA | NA | NA | NA |
| tumor purity score | NA | ns | NA | NA | NA | NA | NA | NA |
| uncharacterized cell | NA | NA | NA | NA | NA | NA | ns | NA |

**Table S8 – *Bacteroides fragilis* impacting specific immune cells across the seven methods that score immune cell infiltration, excluding paired normal samples from the control group.**

NA = Not applicable because the method does not report that type of immune cell. ns = not significant. * = p<0.05, ** = p<0.01, *** p<0.001. Statistical comparison was carried out with Ordinary Least Squares regression to determine which independent variables (sample type, bacterial count, read count) explain the dependent outcome variable (the immune score). Highlighted rows indicate immune cell sub-populations only affected in CRC tissue by *B. fragilis*. A p-value ≤ 0.05 was considered statistically significant.

| Cell_type | Estimate_frac | Estimate_score | Timer | consensus_tme | xcell | mcp_counter | epic | quantiseq |
| --- | --- | --- | --- | --- | --- | --- | --- | --- |
| B cell | NA | NA | ns | ns | ns | ns | ns | NA |
| B cell memory | NA | NA | NA | NA | ns | NA | NA | NA |
| B cell naive | NA | NA | NA | NA | ns | NA | NA | NA |
| B cell plasma | NA | NA | NA | NA | ns | NA | NA | NA |
| Cancer associated fibroblast | NA | NA | NA | ns | ns | ns | ns | NA |
| Class-switched memory B cell | NA | NA | NA | NA | ns | NA | NA | NA |
| Common lymphoid progenitor | NA | NA | NA | NA | ns | NA | NA | NA |
| Common myeloid progenitor | NA | NA | NA | NA | ns | NA | NA | NA |
| cytotoxicity score | NA | NA | NA | ns | NA | ns | NA | NA |
| Endothelial cell | NA | NA | NA | ns | ns | ns | ns | NA |
| Eosinophil | NA | NA | NA | ns | ns | NA | NA | NA |
| Granulocyte-monocyte progenitor | NA | NA | NA | NA | ns | NA | NA | NA |
| Hematopoietic stem cell | NA | NA | NA | NA | ns | NA | NA | NA |
| immune score | NA | ns | NA | ns | ns | NA | NA | NA |
| Macrophage | NA | NA | ns | ns | ns | NA | ns | NA |
| Macrophage M1 | NA | NA | NA | ns | ns | NA | NA | NA |
| Macrophage M2 | NA | NA | NA | ns | ns | NA | NA | NA |
| Macrophage/Monocyte | NA | NA | NA | NA | NA | ns | NA | NA |
| Mast cell | NA | NA | NA | NA | ns | NA | NA | NA |
| MAST cell | NA | NA | NA | ns | NA | NA | NA | NA |
| microenvironment score | NA | NA | NA | NA | ns | NA | NA | NA |
| Monocyte | NA | NA | NA | ns | ns | ns | NA | NA |
| Myeloid dendritic cell | NA | NA | ns | ns | ns | ns | NA | NA |
| Myeloid dendritic cell activated | NA | NA | NA | NA | ns | NA | NA | NA |
| Neutrophil | NA | NA | ns | ns | ns | ns | NA | NA |
| NK cell | NA | NA | NA | ns | ns | ns | ns | NA |
| Plasmacytoid dendritic cell | NA | NA | NA | NA | ns | NA | NA | NA |
| stroma score | NA | ns | NA | NA | ns | NA | NA | NA |
| T cell | NA | NA | NA | NA | NA | ns | NA | NA |
| T cell CD4+ | NA | NA | ns | ns | NA | NA | ns | NA |
| T cell CD4+ (non-regulatory) | NA | NA | NA | NA | ns | NA | NA | NA |
| T cell CD4+ central memory | NA | NA | NA | NA | ns | NA | NA | NA |
| **T cell CD4+ effector memory** | **NA** | **NA** | **NA** | **NA** | ***** | **NA** | **NA** | **NA** |
| T cell CD4+ memory | NA | NA | NA | NA | ns | NA | NA | NA |
| T cell CD4+ naive | NA | NA | NA | NA | ns | NA | NA | NA |
| T cell CD4+ Th1 | NA | NA | NA | NA | ns | NA | NA | NA |
| T cell CD4+ Th2 | NA | NA | NA | NA | ns | NA | NA | NA |
| T cell CD8+ | NA | NA | ns | ns | ns | ns | ns | NA |
| T cell CD8+ central memory | NA | NA | NA | NA | ns | NA | NA | NA |
| T cell CD8+ effector memory | NA | NA | NA | NA | ns | NA | NA | NA |
| T cell CD8+ naive | NA | NA | NA | NA | ns | NA | NA | NA |
| T cell gamma delta | NA | NA | NA | ns | ns | NA | NA | NA |
| T cell NK | NA | NA | NA | NA | ns | NA | NA | NA |
| T cell regulatory (Tregs) | NA | NA | NA | ns | ns | NA | NA | NA |
| tumor purity fraction | ns | NA | NA | NA | NA | NA | NA | NA |
| tumor purity score | NA | ns | NA | NA | NA | NA | NA | NA |
| uncharacterized cell | NA | NA | NA | NA | NA | NA | ns | NA |

**Table S9 – *Fusobacterium nucleatum* impacting specific immune cells across the seven methods that score immune cell infiltration, excluding paired normal samples from the control group.**

NA = Not applicable because the method does not report that type of immune cell. ns = not significant. * = p<0.05. Statistical comparison was carried out with Ordinary Least Squares regression to determine which independent variables (sample type, bacterial count, read count) explain the dependent outcome variable (the immune score). Highlighted rows indicate immune cell sub-populations only affected in CRC tissue by *F. nucleatum*. A p-value ≤ 0.05 was considered statistically significant.
